# Regeneration of Pancreatic Beta Cells by Modulation of Molecular Targets Using Plant-Derived Compounds: Pharmacological Mechanisms and Clinical Potential

**DOI:** 10.3390/cimb45080392

**Published:** 2023-07-26

**Authors:** Clare Njoki Kimani, Helmuth Reuter, Sanet Henriët Kotzé, Christo John Fredrick Muller

**Affiliations:** 1 Biomedical Research and Innovation Platform (BRIP), South African Medical Research Council (SAMRC), Cape Town 7505, South Africa; njokikimani@primateresearch.org; 2 Division of Clinical Pharmacology, Department of Medicine, Faculty of Medicine and Health Sciences, Stellenbosch University, Cape Town 7505, South Africa; 3 Division of Clinical Anatomy, Department of Biomedical Sciences, Faculty of Medicine and Health Sciences, Stellenbosch University, Cape Town 7505, South Africa; 4 Division of Anatomy, Department of Biomedical Sciences, School of Veterinary Medicine, Ross University, Basseterre P.O. Box 334, Saint Kitts and Nevis; 5 Centre for Cardio-Metabolic Research in Africa, Division of Medical Physiology, Faculty of Medicine and Health Sciences, Stellenbosch University, Stellenbosch 7600, South Africa; 6 Department of Biochemistry and Microbiology, University of Zululand, KwaDlangezwa 3886, South Africa

**Keywords:** beta cell, regeneration, plant compounds, plasticity, molecular targets

## Abstract

Type 2 diabetes (T2D) is characterized by pancreatic beta-cell dysfunction, increased cell death and loss of beta-cell mass despite chronic treatment. Consequently, there has been growing interest in developing beta cell-centered therapies. Beta-cell regeneration is mediated by augmented beta-cell proliferation, transdifferentiation of other islet cell types to functional beta-like cells or the reprograming of beta-cell progenitors into fully differentiated beta cells. This mediation is orchestrated by beta-cell differentiation transcription factors and the regulation of the cell cycle machinery. This review investigates the beta-cell regenerative potential of antidiabetic plant extracts and phytochemicals. Various preclinical studies, including in vitro, in vivo and ex vivo studies, are highlighted. Further, the potential regenerative mechanisms and the intra and extracellular mediators that are of significance are discussed. Also, the potential of phytochemicals to translate into regenerative therapies for T2D patients is highlighted, and some suggestions regarding future perspectives are made.

## 1. Introduction

Type 2 diabetes mellitus (T2D) is a major global epidemic with increasing morbidity and mortality linked to its pathophysiology and is, therefore, a serious health burden [1,2,3,4,5]. Despite the introduction of newer drugs in the market, T2D remains a progressive health challenge because these drugs mainly target the clinical symptoms of T2D rather than addressing the underlying pathophysiological factors. Consequently, despite treatment, in the majority of the patients, T2D deteriorates progressively over time, eventually necessitating insulin supplementation [6,7,8]. This progressive clinical deterioration of T2D has inspired the search for new therapeutics that arrest the disease’s progression, protect the remaining pancreatic beta cells, and restore beta-cell mass in T2D by exploiting the inherent plasticity of the pancreas [9,10,11,12,13,14,15].

Although the regenerative potential of the pancreas is reported to be limited in adults [16,17,18,19], several studies have demonstrated plasticity towards beta-cell neogenesis in the adult pancreas following specific physical or physiological stimuli [20,21,22,23]. Mechanistically, this is supported by the loss of transcription factors specific to mature beta cells [24,25,26,27] and the re-expression by beta cells of the pro-endocrine markers Ngn3 and Nanog, suggesting a progenitor-like status [26,27,28]. Further, the plasticity of the pancreas is supported by evidence of pancreatic duct-derived neogenesis, which has been confirmed by using lineage-tracing experiments in mice [29]. In pregnant, obese, insulin-resistant, and diabetic humans, the number of single/small clusters of insulin+ cells and bihormone-expressing cells, and the proportion of insulin+ cells within ducts increase, suggesting that neogenesis may be an important mechanism in adult humans [30,31,32]. Using single-cell RNA sequencing, the existence of multipotent progenitor-like cells within the pancreatic ducts of the human pancreas in patients with T1D and T2D has been confirmed, even after chronic disease [33]. Further, apart from the ductal tree, beta-cell progenitors may also be present in the islets. A “virgin β cell subpopulation” expressing urocortin 3-, MafA-, and insulin+ has been identified in the periphery of the islet as a potential source of progenitor cells that can be reprogrammed toward a beta-cell fate [34]. Additionally, adult mouse islets harbor a population of protein C receptor-positive endocrine progenitors that can be reprogrammed into any of the four endocrine cell types [35].

Interestingly, interventions, including caloric restriction [27], exogenous transcription factor [36] and insulin/glibenclamide therapy [37,38], have been able to reverse beta-cell de-differentiation by restoring the expression of Nkx6.1, Pdx-1 and MafA. Moreover, studies using animal models of DM include alloxan-induced diabetic Wistar rats [39,40,41,42], STZ-induced diabetic Wistar rats [43,44,45], and alloxan-induced diabetic Swiss white mice [46]. In addition, other models for beta-cell regeneration, including partially pancreatectomized Balb/C mice [47] and RIP-CreERT mice crossed with Rosa26-LSL-Lacz mice [48], have been used to show histological evidence of pancreatic architecture recovery and the regeneration of pancreatic islets with a significant increase in the size and number of islets following treatment with herbal remedies compared to diabetic controls, indicating their potential for treatment of T2D. 

Multiple studies have highlighted the antidiabetic effects of plant extracts or their compounds; however, few studies have considered their effects on pancreatic islets or beta cells. Further still, of the studies that have investigated the beta-cell regenerative effects of plant extracts or compounds, for most, the exact mechanism(s) of beta-cell mass recovery have not been fully elucidated [12,49,50,51]. The proposed mechanisms include changes in the transactivation of regulatory genes, transcription factors, cell cycle-related mediators and several downstream signaling pathways. Further, specific small molecules and plant extracts or compounds have been described to induce beta-cell regeneration in vitro. These observations are corroborated in vivo using various models of diabetes through the augmentation of beta-cell mass and the restoration of beta-cell function.

This review highlights studies that have investigated the regenerative potential of plant extracts or plant-derived compounds in animal models or cell-based studies, with an emphasis on the molecular mechanisms that are involved. The eventual goal is to generate a prism through which the common pathways or mechanisms can be identified for possible further scientific inquiry, with the ultimate goal of eventually identifying leads and targets. These could be further validated as either adjunctive to conventional diabetes drugs or as monotherapy for augmenting beta-cell mass and attenuating beta-cell dysfunction, which is characteristic of T2D.

A literature survey was performed in “PubMed” “Scopus”, “EBCOhost”, “Google Scholar” and “Web of Science” using the keywords “anti-diabetic activity”, “beta cell function”, “beta cell proliferation”, “beta cell regeneration”, “islet regeneration”, “polyphenols”, “plant extracts”, “plant compounds”, and “beta cell differentiation” to evaluate the effects of each plant product. To investigate the effects of natural products on pancreatic beta-cell regeneration in diabetes, we included articles describing the effects of crude plant extracts or plant-derived compounds on beta-cell function using cell cultures, isolated rodent islets, diabetic animal models and human islets derived from cadaveric donors. We summarized all research articles highlighting the mechanistic potential of plant extracts or compounds on molecular markers of beta-cell proliferation, de-differentiation, transdifferentiation and neogenesis. 

## 2. Phytotherapy and Regeneration of Pancreatic Beta Cells 

**Figure 1:**
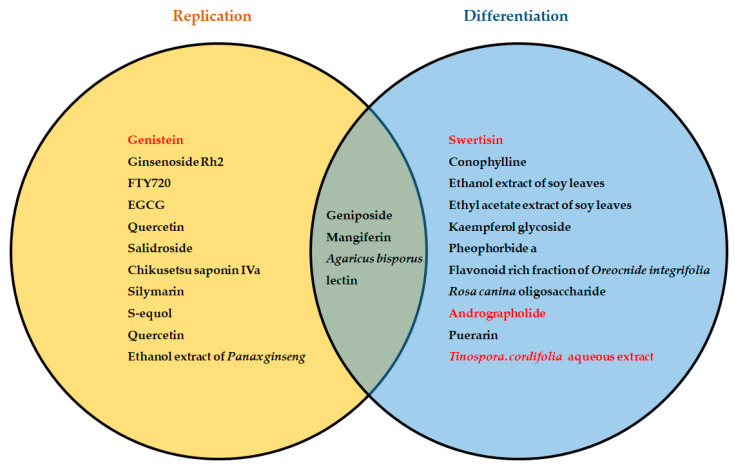
Plant extracts/phytocompounds mediate beta-cell regeneration via the replication of beta cells or the differentiation of progenitor cells or other islet cell types into functional beta cells. Compounds/extracts highlighted in red have been tested in human islets or ductal cells.

Due to the chronic progressive nature of T2D, there have been persistent efforts to identify compounds that can stimulate beta-cell regeneration and prevent apoptosis, leading to a return of endogenous control of glucose homeostasis [12,49,50,51,52,53,54]. Various plant extracts and/or plant-derived compounds have been shown to mediate beta-cell regeneration by modulating the transcription and translation of beta-cell-specific transcription factors and cell cycle regulatory proteins to enhance cell replication, differentiation and neogenesis. The regenerative effects of these extracts or compounds are mostly attributed to the presence of different classes of flavonoids that include flavon-3-ols, flavanones, anthocyanins, flavones and isoflavones. Certain alkaloids have also been shown to possess beta-cell regenerative activity [12,49,50,51]. Figure 1 summarizes the regeneration mechanisms of the various plant extracts or compounds. Table 1 summarizes beta-cell regenerative mechanisms based on the in vitro, ex vivo and in vivo studies. The cellular proliferation of remnant beta cells is critical to the increase in beta-cell mass under physiological and pathophysiological stress conditions. Replication has been identified experimentally to play a key role in various models of experimental diabetes. In the context of plant extracts or compounds, cell cycle regulatory proteins are amenable to alteration to effect enhanced beta-cell proliferation. Neogenesis, or the differentiation of beta cells from non-beta-cell progenitors, is another key mechanism for the formation of new beta cells. This is dependent on the expression of markers for stem/progenitor cells. In the case of beta-cell differentiation from fully differentiated non-beta cells, poly-hormonal cells, or cells expressing markers for multiple pancreatic cell types, are key features. Various plant extracts and compounds have displayed a capacity to activate neogenetic processes in different experimental models of diabetes. 

## 3. Extracellular Signaling Pathways That Mediate Phytochemical Induced Beta-Cell Regeneration 

**Figure 2:**
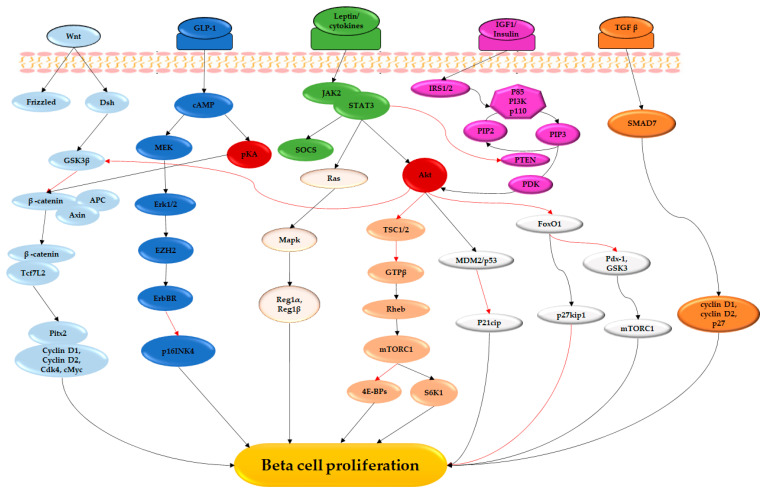
Signaling pathways that mediate phytochemical-induced beta-cell proliferation. The black and red arrows represent upregulation/activation and inhibition, respectively.

Several extracellular signal molecules and intracellular signaling pathways have been shown to underlie islet plasticity in situations of beta-cell stress and dysfunction. These signaling pathways mediate their effects via activation or repression of the transcription factors that mediate beta-cell proliferation and survival. Several endogenous beta-cell mitogens that have been identified include glucose, insulin, growth hormone, epidermal growth factor (EGF), gamma-aminobutyric acid (GABA), glucagon-like peptide (GLP-1), glucose-dependent insulinotropic polypeptide (GIP), hepatocyte growth factor (HGF) and SerpinB1. Although intracellular pathways that mediate beta-cell regeneration have been documented, the full extent of their interactions has not been fully elucidated. Further, given that most studies have been done either in vitro or in vivo studies using rodent models, their translatability in the human pancreas remains to be established [14,84,85]. A summary of the signaling pathways that mediate beta-cell proliferation due to plant compounds is described in the subsequent sections and summarized in Figure 2.

Multiple signaling pathways have been shown to stimulate beta-cell regeneration by modulating the transactivation of transcription factors for beta-cell development and cell cycle control proteins. The pathways illustrated have been described in rodent pancreas, and it is not clear whether the same pathways are involved in beta-cell regeneration in humans. The Wnt/frizzled pathway phosphorylates GSK-3β, which inhibits the phosphorylation of β-catenin, thus activating the transcriptional activity of Tcf7L2. Tcf7L2 mediates the transcription of Pitx2 and downstream targets cyclin D1, cyclin D2, cdk4 and cMyc, activating the cell cycle and enhancing proliferation. On binding to its receptor, GLP-1 activates cAMP-PKA, leading to the phosphorylation and activation of the MEK1 and MEK2, which then phosphorylate and activate the MAP kinases Erk1/2. GLP-1 signaling via pKA interacts with the Wnt pathway by phosphorylating β-catenin. The binding of insulin and IGF-1 to their respective receptors induces the phosphorylation of serine and threonine residues on IRS1/2, resulting in the activation of PI3K and Akt. Akt phosphorylates and inactivates downstream targets GSK-3β and FoxO1, thereby inhibiting their antiproliferative effects. Further, downstream of Akt, MDM2/p53 inhibits p21Cip1 activity, enhancing cell proliferation. Similarly, activation of the JAK-STAT pathway inhibits PTEN and also activates Akt signaling. Activation of the JAK2/STAT3 signaling also induces SOCS proteins, which have a role in the temporal control of the JAK2/STAT3 pathway. Ellipses of the same color depict signals within the same pathway. The black and red arrows represent upregulation/activation and downregulation/inhibition, respectively. 

Dsh: dishevelled; GSK-3β: glycogen synthase kinase-3β; APC: adenomatous polyposis colis; Tcf7L2: Transcription factor 7-like 2; Cdk4: cyclin-dependent kinase 4; GLP-1: glucagon-like peptide 1; cAMP: cyclic adenosine monophosphate; pKA: protein kinase A; MEK: mitogen-activated protein kinase kinase; Erk1/2: extracellular signal-regulated kinase; EZH2: enhancer of zeste homolog 2; JAK-STAT: janus kinase-signal transducers and activators of transcription; SOCS: suppressor of cytokine signaling; IGF-1: insulin-like growth factor 1; IRS: insulin receptor substrate; PI3K: phosphoinositide 3-kinase; PIP2: phosphatidylinositol (4,5)-bisphosphate; PIP3: phosphatidylinositol (3,4,5)-trisphosphate; PDK1: phosphoinositide-dependent kinase 1; PTEN: phosphatase and tensin homolog; TSC1/2: tuberous sclerosis complex 1/2; Rheb: Ras homolog enriched in brain; mTORC1: mechanistic target of rapamycin complex; 4E-BP: eukaryotic translation initiation factor 4E-binding protein 1; S6K1: ribosomal S6 kinase 1; MDM2: mouse double minute 2 homolog; FoxO1: forkhead box protein O1; Pdx-1: pancreatic and duodenal homeobox 1; TGFβ1: transforming growth factor β1; Ras: rat sarcomas. Modified with permission [14].

**Figure 3:**
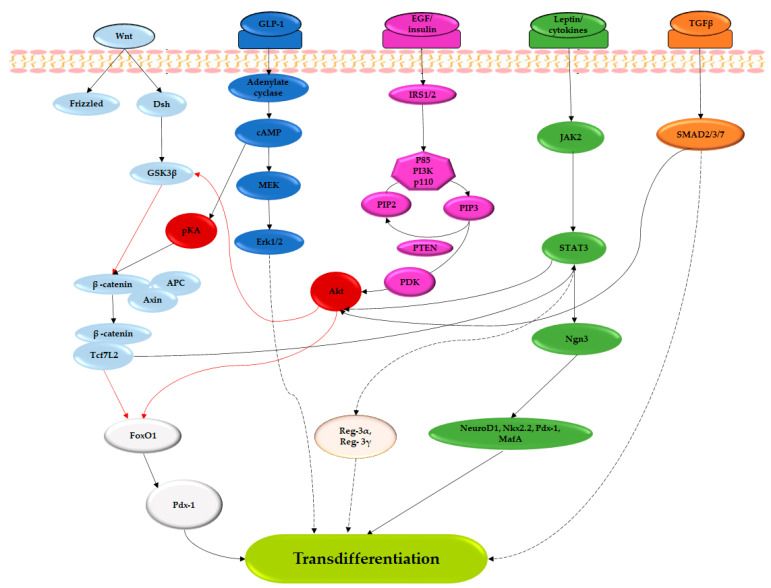
Summary of molecular signals underlying the transdifferentiation of acinar or ductal cells into insulin-secreting beta-like cells. Ellipses of the same color depict signals within the same pathway. Black and red lines represent upregulation/activation and inhibition, respectively. The black and red arrows represent upregulation/activation and downregulation/inhibition, respectively. The dashed lines represent pathways where downstream intermediates are unknown/unclear.

In this review, the term neogenesis has been broadly used to refer to the generation of beta cells from non-beta cell precursors, including fully differentiated progenitor cells and non-beta cell pancreatic cells. The delineation between neogenesis and transdifferentiation can be confusing because studies have identified progenitor cells within the pancreatic ductal structures, while duct cells have also been shown to differentiate into insulin-producing beta-like cells following reprograming using a cocktail of Pdx-1, Ngn3 and MafA transcription factors (Spears et al., 2021). However, in the studies summarized in the current review, geniposide [64], *T. cordifolia* [83], *O. integrifolia* [78], puerarin [79], swertisin [47], conophylline [58] and andrographolide [56] mediated their beta-cell regenerative effects via the transdifferentiation of acinar or ductal cells into beta/beta-like cells. Transdifferentiation of these cells into beta cells was associated with a decreased expression of carbonic anhydrase 9 and an increased expression of pro-endocrine Ngn3, nestin, neuroD1, Nkx2.2, Pdx-1 and MafA [47,56,58,64,78,79,83]. This expression of pro-endocrine markers and the loss of ductal cell differentiation marker carbonic anhydrase 9 [83] supports the initial de-differentiation of these cells into a progenitor-like status and thereafter, the differentiation of these progenitor-like cells into beta-like endocrine cells. Additionally, the upregulated expression of GLP-1R [79], SMAD proteins [47], Wnt/B-catenin, TCF7L2, JAK2 and STAT3 [64,79] implies their role in mediating this conversion. Previous studies have also reported the involvement of JAK2/STAT3 [86,87,88], Wnt/TCF7L2 [88], GLP-1R [88] and EGFR/ERK1/2 [89] signaling in the transdifferentiation of acinar/ductal cells into beta cells. However, the downstream mediators for transdifferentiation in these pathways need further elucidation (Figure 3). Although human exocrine and ductal cells can successfully express endocrine pancreatic markers and secrete insulin in vitro following treatment with plant extracts or compounds [47,56,83], the potential for human exocrine and ductal cells to significantly contribute to beta-cell regeneration in vivo would be difficult to estimate due to the requirement of lineage tracing.

Dsh: dishevelled; GSK-3β: glycogen synthase kinase-3β; APC: adenomatous polyposis colis; Tcf7L2: Transcription factor 7-like 2; Cdk4: cyclin-dependent kinase 4; GLP-1: glucagon-like peptide 1; cAMP: cyclic adenosine monophosphate; pKA: protein kinase A; MEK: mitogen-activated protein kinase kinase; Erk1/2: extracellular signal-regulated kinase; JAK-STAT: janus kinase-signal transducers and activators of transcription; EGF-1: Epidermal growth factor; IRS: insulin receptor substrate; PI3K: phosphoinositide 3-kinase; PIP2: phosphatidylinositol (4,5)-bisphosphate; PIP3: phosphatidylinositol (3,4,5)-trisphosphate; PDK1: phosphoinositide-dependent kinase 1; PTEN: phosphatase and tensin homolog; TGFβ: transforming growth factor beta; Ngn3: neurogenin 3; Pdx-1: pancreatic and duodenal homeobox 1; MafA: musculoaponeurotic fibrosarcoma oncogene A; Reg-3α/γ: regenerating islet-derived 3-alpha/gamma. Modified with permission [14].

### 3.1. PI3K/Akt Signaling Pathway 

The Akt signaling pathway is one of the major pathways involved in the maintenance of beta-cell mass [90]. The key ligands in this pathway are insulin and insulin-like growth factor (IGF), glucose, GLP-1 and GIP. Stimulation of the GLP-1, GIP, IGF and insulin receptors, which possess intrinsic tyrosine kinase activity, leads to the phosphorylation of tyrosine residues in insulin receptor substrate proteins (IRS1 and IRS2), leading to their activation. The activation of IRS uncovers binding sites for PI3K, leading to its activation and phosphorylation of phosphatidylinositol-4,5-biphosphate (PIP2) to form phosphatidylinositol-3,4,5-triphosphate (PIP3). PIP3 then recruits Akt and phosphoinositide-dependent kinase-1 (PDK1) to the membrane. Here, PDK1 phosphorylates Akt at Thr308, while mTORC2 phosphorylates Akt at Ser473 [91]. Subsequently, Akt is translocated to the nucleus, where it phosphorylates various target substrates that modulate the beta-cell function and proliferation status. The activity of Akt is regulated by PTEN, which dephosphorylates PIP3; moreover, protein phosphatase 2A and PH domain leucine-rich repeat protein phosphatase inactivate Akt by dephosphorylating Thr308 and Ser473, respectively [92,93]. Furthermore, Akt activity can also be inhibited by JNK, which phosphorylates IRS1 on Ser307, thereby decreasing the interaction between IRS1 and the insulin receptor (IR) [94]. This explains why activation of the JNK pathway in the pathogenesis of diabetes results in the inhibition of Akt signaling via the suppression of IR signaling [95,96]. Activation on the JNK signaling pathway inhibits the ability of Pdx-1 to bind to DNA in beta cells [97]. Furthermore, mTOR/S6K signaling also inhibits Akt signaling by phosphorylating IRS1 at Ser302, 307, 636 and 639 [98]. 

The Akt signaling pathway plays a critical role in beta-cell proliferation and survival in and of itself as well as via the intricate associations with various other molecules and signaling pathways, including the FoxO1, GSK3β, mTOR, and GLP-1 signaling and epidermal growth factor (EGF) signaling pathways [14,85]. The interactions between Akt and the ERK1/2 signaling pathways upregulate Pdx-1 expression by inhibiting FoxO1 [85]. Aside from binding to the GLP-1R, GLP-1 transactivates the EGF receptor (EGFR) and c-Src, resulting in PI3K activation [99]. Moreover, GLP-1 induces cAMP and the cAMP response element-binding (CREB)-dependent transcription of IRS2 [100]. Exendin-4, a GLP-1 analog, induces IRS2/Akt signaling in mice, resulting in an increased beta-cell mass, which suggests a prominent role for IRS2 in GLP-1-mediated signaling [101]. In 60% pancreatectomized rodents, the proliferation of ductal cells was shown to be mediated by Akt signaling via the induction of CREB and FoxO1 [102]. Studies have also shown that mTOR, via its downstream molecules hamartin (TSC1), tuberin (TSC2) and Rheb, interact with the Akt pathway [98,103]. Following the cues from growth factors or nutrients, mTORC1, via the phosphorylation of eukaryotic initiation factor 4E-binding protein 1 (4EBP1) and S6K, induces cell proliferation [104]. Following the phosphorylation of 4EBP1 by mTOR, eIF4E is released from 4EBP, leading to mRNA translation [105]. The role of mTORC1 in regulating Akt-mediated beta-cell proliferation is confirmed in studies using mice where rapamycin inhibited Akt-induced beta-cell proliferation by inhibiting the expression of cyclin D2 [106]. Similarly, rapamycin treatment inhibited glucose-induced beta-cell proliferation by inhibiting thymidine incorporation [107]. Meanwhile, the deletion of Tsc2 in mouse beta cells resulted in the attenuation of diabetic symptoms and an increase in beta-cell proliferation [108,109]. Conversely, upstream of Akt, mTORC2 has been shown to regulate Akt signaling via phosphorylation at Ser473 [110]. 

Another key target of Akt signaling is Pdx-1. Using transgenic mice engineered to constitutively express active Akt in beta cells, Jara et al., 2020 [111] showed that Akt also induced beta-cell expansion, an effect that was mediated by Pdx-1. Moreover, Pdx-1 haplosufficient mice experienced decreased beta-cell proliferation [111]. Similar studies have indicated that Akt regulates the translation of cyclinD1, cyclinD2, p21 and Cdk4 activity, thereby underpinning the role of the Akt pathway in the regulation of beta-cell proliferation [112,113]. Another pathway that interacts with the Akt pathway is the particulate guanylyl cyclase/cyclic guanidine monophosphate/protein kinase G type Iα (sGC/cGMP/PKG) pathway. Knockdown of protein kinase G type Iα in beta cell lines suppressed cell proliferation and survival due to a decreased expression of Akt, phospho-FoxO1 and Pdx-1 [114]. Similarly, INS-1 beta cells and pancreatic islets that were exposed to atrial natriuretic peptide (ANP), a hormone that activates protein kinase G (PKG) signaling, experienced enhanced cell proliferation that was mediated by PKG’s activation of the PI3K/p-Akt/p-FoxO1/cyclin D2 signaling pathway [115]. Akt signaling also mediates beta-cell survival by inhibiting apoptotic signals. INS1 cells that overexpressed the active form of Akt were resistant to palmitate-induced ER stress and apoptosis [116,117]. Akt also inhibits JNK signaling, thus protecting the cells against oxidative stress and cytokine-induced damage [118,119].

Glycogen synthase kinase-3β (GSK-3β), a serine/threonine kinase, also has a role in modulating beta-cell proliferation. Akt phosphorylates GSK-3β on serine 9, leading to its inactivation [92]. Additionally, GSK-3β phosphorylates β-catenin, thereby targeting its ubiquitination and proteosomal degradation [120]. Further, GSK-3β phosphorylates cyclin D1, targeting its degradation [121]. GSK-3β also regulates the Wnt signaling pathway upstream of β-catenin; moreover, GSK-3β regulation of cyclin D1 expression is dependent on β-catenin [121,122,123,124]. Hyperphosphorylation of GSK-3β led to an increased expression of cyclin D1 in mouse islets, suggesting a direct role for GSK-3β in regulating beta-cell proliferation [112], mediated by the effects of Akt. Cyclin D1 is the key cyclin for initiating cell cycle progression in beta cells [125,126]. Mice with a reduced expression of the insulin receptor had lower phospho-GSK-3β levels and were more susceptible to ER stress and apoptosis [127]. GSK-3β haplosufficient mice that were lacking the Irs2 gene had a mitigated diabetic phenotype that was characterized by enhanced beta-cell proliferation, increasing the expression of Pdx-1 and suppression of p27 [128]. Conversely, GSK-3β knockdown [117] or the inactivation of GSK-3β using small molecules [121] protects the beta cell against apoptosis and the induced proliferation of INS1 beta cells [121], indicating the key role of the PI3K/Akt/GSK-3 pathway in beta-cell survival and proliferation. Indeed, GSK-3β has been shown to instigate the translocation of Pdx-1 from the nucleus to the cytoplasm and decrease the expression of cyclin D1 [129].

FoxO1 is another downstream target of Akt. Akt phosphorylates FoxO1, leading to its translocation from the nucleus to the cytoplasm, thus inhibiting its transcriptional activity. Because FoxO1 has been shown to inhibit cell proliferation by inhibiting the cyclin/cdk complexing and upregulating the expression of p27, p21, p15 and p19 [130], its phosphorylation by Akt releases this inhibitory effect, thereby promoting cell proliferation. Further, inhibition of FoxO1 has been shown to enhance beta-cell mass via an increased expression of Pdx-1 and cyclin D1 and a decreased expression of p27 in mice that were deficient for either Irs2 or PDK1, thereby implicating FoxO1 as a downstream target of the Akt pathway [131,132,133]. Further, in conditions of insulin resistance, FoxO1 prevents the adaptive increase in beta-cell mass [134,135]. Glucose, or GLP-1-induced nuclear-cytoplasmic shuttling of FoxO1, is mediated by PI3K/Akt signaling and EGFR activation [136,137].

Downstream of the Akt pathway, ribosomal S6 kinase (S6K) is activated by phosphorylation at Thr 389 by mTOR and phosphorylation at the T-loop by PDK1 following stimulation by multiple stimuli, including insulin and growth factors. Subsequent to S6K activation, the 40S ribosomal protein S6 is phosphorylated, which culminates in increased translation of a variety of mRNA that encode ribosomal proteins and translation elongation factors that are critical for cellular growth and proliferation [103,138]. S6K-deficient mice experience glucose intolerance, characterized by a reduced beta-cell size [138]. S6K is a negative regulator of Irs1 and Irs2, which inhibits Akt signaling [98].

Akt has been shown to enhance beta-cell proliferation by activating the cyclin D/cdk4 complex [112] and via the phosphorylation of cell cycle inhibitors p21 and p27, leading to their sequestration in the cytoplasm and subsequently proteosomal degradation [130], thereby abolishing their inhibition of cdk activity and enhancing cell cycle progression. PI3K/Akt signaling may also regulate cell cycle progression by inhibiting the transcription and translation of cell cycle inhibitors [130]. In this sense, Akt signaling has been associated with increased beta-cell mass via proliferation and neogenesis and increased beta-cell size in mice overexpressing constitutively active Akt in beta cells [139].

### 3.2. Transforming Growth Factor β Pathway 

Transforming growth factor β (TGFβ) belongs to a signaling superfamily that modulates pancreatic development and cellular specification and has been linked to beta-cell regeneration after pancreatic injury [140]. TGFβ ligands (TGFβ 1–3) bind to TGFβ type II receptors on the cell membrane, leading to the phosphorylation and activation of TGFβ type I receptors. Consequently, Smad2 and Smad3 are phosphorylated, after which they complex with Smad4. The Smad2/3/4 complex translocates to the nucleus, where it initiates transcriptional activity. Smad7 inhibits the phosphorylation of Smad2/3, thereby inhibiting TGFβ signaling [141,142].

Studies using adult human islets showed that TGFβ stabilizes the beta-cell phenotype by preventing its differentiation into duct-like structures. Further, TGFβ signaling prevents beta-cell apoptosis. However, as these effects of TGFβ occur alongside a dysregulated GSIS response, TGFβ appears to negatively affect beta-cell function [143]. The effects of TGFβ signaling appear to depend on the cell type and on the physiological environment [144]. Indeed, Sjoholm and Hellerstrom showed that the effect of TGFβ signaling on beta-cell function and proliferation is dependent on the glucose concentration [145]. Moreover, while Smad7 induces beta-cell proliferation in the PDL and ppx models of diabetes, the overexpression of Smad7 in beta cells induces a reversible diabetic phenotype [146].

Isolated human islet cells exposed to a redifferentiation cocktail containing exendin-4 and activin A demonstrated the upregulation of TGFβR1, Smad2 and TGFβ2 expression as well as an increased activation of the Smad complex and its subsequent nuclear localization. However, when the TGFβ pathway was blocked using short hairpin RNA against TGFβR1 mRNA, cell proliferation, beta-cell-derived cell de-differentiation and epithelial-to-mesenchymal transition were inhibited [147]. Meanwhile, the mRNA expression of IAPP, insulin and beta-cell-specific transcription factors was upregulated with an increase in C peptide-positive cells and the induction of a mesenchymal-to-epithelial transition [147]. This study thus shows that the repression of the TGFβ pathway maintains the beta-cell differentiated state. Other studies supporting these findings indicate that Smad3 inhibits MafA transcriptional activity in human and mouse cells via direct interaction with MafA [148]. Moreover, Smad3 occupies the insulin promoter, thereby inhibiting insulin synthesis [149]. A similar role for TGFβ signaling has been established, observing inhibition of HIT-T15 pancreatic beta-cell proliferation by TGFβ signaling. Conversely, TGFβ inhibition promoted beta-cell proliferation [150]. This proliferative effect was associated with the decreased protein expression and decreased sequestration of the Cdk inhibitor, p27, in the nucleus following TGFβ signaling inhibition [150].

A study by Sehrawat et al., 2020, shows that the beta-cell-specific overexpression of Smad7 in mice augments beta-cell proliferation without affecting beta-cell function. Although some beta-cell-specific transcription factors were downregulated following Smad7 overexpression, NeuroD1 expression was upregulated, potentially helping to maintain normal beta-cell function [151]. Beta-cell de-differentiation, associated with increased Smad7 expression, has been confirmed in other studies, suggesting that this may be a temporary phase prior to proliferation and subsequent redifferentiation [141]. The TGFβ pathway may also mediate beta-cell de-differentiation via its direct interaction with the Notch signaling pathway. TGFβ signaling has been shown to upregulate the expression of Hes-1, which is a downstream Notch target. Here, Smad3 and the intracellular domain of Notch 1 (NICD) were shown to interact directly [152]. Similar to TGFβ signaling, Notch signaling is associated with beta-cell de-differentiation, repressed MafA expression, enhanced MafA degradation, impaired beta-cell function and enhanced proliferation of dedifferentiated cells [153]. Smad7 has also been associated with beta-cell regeneration, observed in adult 60% ppx [154] and PDL [155] diabetic rodent models. In the PDL model, Smad7-mediated beta-cell proliferation is mediated by the upregulation of cyclins D1 and D2 and the translocation of p27 from the nucleus to the cytoplasm [156]. A similar mechanism of TGFβ signaling induced beta-cell proliferation via Smad7, through the exclusion of p27, from the nucleus, has been described [146]. Aside from this direct effect, Smad7 also mediates the interaction of the TGFβ signaling pathway with other signaling pathways, including MAPKs and the inflammatory pathways. TGFβ signaling was shown to increase phosphorylation of Erk, p38 and Smad2 and decrease JNK phosphorylation [143].

A study by Hayes et al., 2016, identified inhibin beta-B (Inhbb), which is a subunit of activin and a member of the TGFβ superfamily, as a direct Pdx-1 target and a key effector of Pdx-1-mediated beta-cell proliferation in rat and human islets, further providing mechanistic insight into the beta-cell proliferative role of Pdx-1. The proliferative effect of Pdx-1 was mediated by the ability of Inhbb to increase the expression of cyclin D1 [157]. The beta regenerative importance of TGFβ signaling was recently highlighted in a study by Wang et al., 2019 [158], which showed a synergistic interaction between the TGFβ signaling pathway and harmine, a dual-specificity tyrosine-regulated kinase-1a (DYRK1A) inhibitor, resulting in significantly increased beta-cell replication rates of 5%–8%, leading to increased beta cell numbers in human and mouse models.

### 3.3. GLP-1 Signaling and Beta-Cell Regeneration

Glucagon-like peptide 1(GLP-1) is one of the incretin hormones that is produced by the L cells of the small intestines following the intake of carbohydrates and fat [159]. GLP-1 has been shown to mediate beta-cell proliferation in both in vitro and in vivo models. Buteau et al., 2003 [99] established that GLP-1 induced beta-cell proliferation via the c-Src-dependent cleavage of BTC from the membrane, transactivation of EGFR and downstream activation of PI3K signaling. Similarly, other studies support increased PI3K activity, increased Pdx-1 expression and DNA binding activity as events downstream of GLP-1 mediated beta-cell proliferation [160]. Downstream of PI3K, GLP-1 signaling upregulates the expression of ERK1/2, p38 MAPK and Akt, corresponding to the proliferative response to GLP-1 [161]. Furthermore, GLP-1 signaling, through its downstream targets PI3K and PDK, activated PKCζ, enhancing its translocation to the nucleus [161]. Pdx-1, which has been associated with beta-cell proliferation, is a downstream target of PKCζ. [162]. Moreover, mechanistic studies have identified Akt as an obligatory downstream mediator of GLP-1-induced beta-cell proliferation [163,164], suggesting that p38 MAPK and PKCζ potentially act downstream of Akt.

Although the mitogenic effects of GLP-1 or its analogs have been documented for both the in vivo and in vitro models, these effects may vary depending on the model. For example, GLP-1 is mitogenic in *db*/*db* and C57Bl/6 mice [165,166] but does not seem to mediate any proliferative effects in the 70% ppx Balb/c mice [167]. Mechanistically, GLP-1 may mediate its beta-cell regenerative effects via both neogenesis and beta-cell replication. In 4–5-week-old Sprague-Dawley rats, treatment with the GLP-1 analog exendin-4 induced the differentiation of duct cells into islet cells. These duct cells were found to express the GLP-1 receptor—furthermore, exendin-4 enhanced beta-cell replication [168]. Similarly, in neonatal STZ-induced diabetic Wistar rats, GLP-1 and exendin-4 enhanced beta-cell mass via the differentiation of ductal precursors and augmented beta-cell proliferation [169]. This confirms that GLP-1 signaling mediates beta-cell proliferation by inducing both neogenesis and beta-cell replication.

### 3.4. Wnt/β-Catenin Signaling and Beta-Cell Regeneration

The Wnt signaling pathway is a key regulator of cellular proliferation, the determination of cell fate and differentiation in multiple organs, including the pancreas [170]. The canonical Wnt pathway is activated when the Wnt ligands bind to the Wnt Frizzled and LRP5/6 co-receptors. Dishevelled protein (Dvl)I is activated, which inhibits GSK-3β and releases β-catenin from its degradation complex, resulting in its stabilization, dephosphorylation, and cytoplasmic accumulation and translocation into the nucleus. Here, β-catenin interacts with members of the TCF/LEF family of transcription factors, which include TCF7L2 inducing the transcription of target genes [170,171]. In vitro studies show that the addition of Wnt3a upregulates the expression of Pitx2, cyclin D1, cyclin D2 and cdk4 in MIN6 cells and in purified human and mouse islets and beta cells [123], leading to enhanced beta-cell proliferation. Pitx2 mediates its effects by promoting the transcription of cyclin D1, cyclin D2 and c-myc and by repressing the inhibitory action of histone deacetylase 1 and the pocket protein p130 [171,172]. β-catenin, an effector molecule downstream of Wnt, displays similar mitogenic effects in vivo [123]. Further mechanistic interrogation suggests that the Wnt signaling pathway interacts with the GLP pathway. GLP-1 and its analog, exendin-4, were shown to activate Wnt signaling in INS-1 cells and isolated islets via the GLP-1R [173]. Exendin-4-augmented-Wnt signaling was mediated by the downstream targets Akt and GSK-3β. Moreover, exendin-4-induced-beta-cell proliferation was predicated on the activation of the Wnt signaling pathway [173]. Wnt signaling was dependent on active Akt and inactivated GSK-3β. Meanwhile, GLP-1 and the exendin-4 activation of Wnt signaling was mediated by cAMP-dependent pKA, Akt and ERK1/2 activation downstream of the GLP1R. This effect is independent of the activation of PI3K and EGFR and/or the CREB or GSK-3 statuses [173]. Furthermore, pKA phosphorylates β-catenin on Ser675, which inhibits its ubiquitination and promotes its complexing with TCF7L2 and subsequent gene transcription [174]. Exendin-4 was found to induce the transcription of the cell cycle-initiating protein cyclin D1 by augmenting the interaction of TCF7L2 and β-catenin with the cyclin D1 promoter [173]. Other studies [175] found an increased expression of Wnt4, a signaling molecule in the canonical Wnt pathway, further corroborating the GLP-1/Wnt signaling pathway interplay. Similarly, in the NIT-1 clonal beta-cell line, Wnt3a treatment achieved augmented cell proliferation that was mediated by both Wnt/β-catenin signaling and increased IRS2 expression and IRS2 phosphorylation, as well as Akt phosphorylation and Akt-mediated phosphorylation of GSK-3β, suggesting crosstalk between the Wnt/β-catenin and IRS2/PI3K/Akt signaling pathways [176].

The Wnt signaling pathway is also involved in mediating beta-cell hyperplasia in the compensatory phase of T2DM [88,177]. Aside from mediating beta-cell regeneration via enhancing beta-cell proliferation, the Wnt signaling pathway has been shown to induce beta-cell neogenesis from the ductal progenitor cells in neonatal mice via the downregulation of GSK-3β [178], while overexpression of TCF7L2 in human exocrine tissues induced the formation of islet-like cell clusters within the periphery of the duct cells in a JAK2/STAT3-dependent mechanism [88]. JAK2/STAT3-mediated exocrine to beta-like cell reprograming was also associated with an increased expression of the pro-endocrine TFs HNF-6 and Ngn3 and beta-cell factor Pdx-1 [88].

### 3.5. JAK2/STAT3 Signaling in Beta-Cell Regeneration

One of the other pathways that have been documented to mediate phytochemical-induced beta-cell regeneration is the JAK2/STAT3 signaling. Signal transducers and activators of transcription-3 (STAT3) proteins regulate the cell proliferation of multiple cell types [179]. Upon binding to their cognate receptors, ligands, which include various cytokines, EGF, VEGF and leptin, induce the phosphorylation of Janus kinases (JAK); subsequently, phosphorylation of the receptor tails recruits STAT proteins, which are then phosphorylated. The activated STAT proteins dimerize and translocate to the nucleus, where they regulate transcription [180]. Valdez et al., 2016 [181] reported that inflammatory cytokines activate STAT3, increasing the beta-cell proliferation and numbers by stimulating the epithelial-to-mesenchymal transition and reprograming mouse and human pancreatic ductal PANC-1 cells to beta cells via the upregulation of Ngn3 expression. Similarly, Baeyens et al., 2006 [86] showed that beta-cell neogenesis from adult acinar tissue is mediated by the JAK2/STAT3 signaling pathway via the upregulated expression of Ngn3. Moreover, in STAT3-transduced dispersed rat islets, there was a significant increase in proliferation [179], and in 1.1B4 cells, a human beta-cell line, the transcription of the REG Iα and REG Iβ genes that are induced during beta-cell regeneration, was regulated by JAK2/STAT3 signaling [182]. In contrast, suppression of STAT3 signaling with concomitant exogenous expression of the transcription factors Pdx-1, Ngn3 and MafA augmented reprogramming of the mPAC and mouse acinar cells into beta cells [183]. The disparities in the effects of STAT3 signaling in the mentioned studies may be a result of timing and the degree of STAT3 activation [183].

## 4. Conclusions and Future Perspectives: Potential of Phytochemicals as Mediators of Beta-Cell Regeneration

This review explored the beta-cell regenerative effects of plant extracts and compounds with a focus on the regenerative mechanisms involved. Primarily, two major mechanisms appear to be involved. The first is the generation of new beta cells from non-beta cell precursors or from stem/progenitor cells. The second mechanism is through the enhancement of cellular proliferation of pre-existing beta cells. Given the scope of the studies covered in this review, cellular proliferation appears to be more amenable to activation by plant extracts/compounds. Nevertheless, studies have indicated that regenerative mechanisms or regenerative capacity are dependent on several factors.

Firstly, islet age appears to play a significant role, and although both young and aged islets have been shown to possess regenerative potential, this capacity declines with age, and younger islets display a greater capacity for regeneration [184]. Consequently, plant compounds have a greater regenerative effect in embryonic pancreata or neonatal pancreas [59,60,61] by inducing both neogenesis and enhancing beta-cell proliferation. The type of diabetic model may also alter the dynamics of plant compound-derived beta-cell regeneration. Indeed, in adult STZ-induced type I diabetic rodent models, beta-cell proliferation appears to be the predominant regenerative mechanism [65,67,80]. However, this effect appears to be dependent on the age of the animal [59,61]. Conversely, in type 2 diabetic models, *db*/*db* mice [66,185] and HFD-fed C57BL/6J mice [64,66,68], both neogenesis and beta-cell proliferation were responsible for beta-cell regeneration.

The severity of pancreatic injury, dysfunction or beta-cell deficiency also appears to determine the attendant beta-cell regenerative mechanisms [186,187]. Thus, following pancreatectomy, the regeneration of beta cells occurs essentially via the replication of existing beta cells [16,188,189,190,191,192,193]. However, a few studies have reported neogenesis in 60% ppx mice [20,192]. Conversely, after 90% ppx, beta-cell regeneration is mediated by both neogenesis and the replication of remnant beta cells [191,193,194,195]. Essentially, while 90% ppx causes hyperglycemia, 50–70% ppx does not, and given that hyperglycemia induces beta-cell proliferation, it is anticipated that the hyperglycemic state in 90% ppx provides an additional stimulus for beta-cell neogenesis beyond that which is requisite for the replication of existing beta cells [196]. A partial pancreatectomy of >70% in rodents is an experimental model of severe pancreatic injury. In 70% of the pancreatectomized mice, a flavonoid-rich fraction of *Oreocnide integrifolia* [78], mangiferin [77] and swertisin [47] induced beta-cell neogenesis and beta-cell proliferation. Severe beta-cell damage has been suggested to activate more robust beta-cell regenerative mechanisms [187].

Various in vitro models exist for the study of beta-cell regenerative activity. These include several types of pancreatic beta cell lines, including BTC3, INS-1, MIN-6, RIN-5F, human beta cells and isolated islets. In these beta-cell models, cellular proliferation appears to be the exclusive mechanism of regeneration following exposure to plant compounds [57,65,67,71,73,197]. Conversely, in the non-beta-cell in vitro models, including AR42J-B13, a rat pancreatic cell line of exocrine origin [58], NIH3T3 [74] and human pancreatic ductal cells (PANC-1) [47], plant compounds activate neogenetic activity, resulting in the formation of functional islet-like cell clusters whilst maintaining a population of the progenitor cells via increased proliferation.

Although the mechanisms that underlie the observed beta-cell regeneration due to plant extracts/compounds are still not fully elucidated, they appear to involve the modulation of beta-cell differentiation transcription factors and cell cycle regulatory proteins and suggest the involvement and interaction of multiple signaling pathways. Interestingly, beta-cell regeneration appears to be mediated primarily via the PI3K/Akt/FoxO1 signaling pathway [63,66,69,73,197] and, to a lesser extent, the cAMP/PKA pathway [65,67]. Conversely, the SMAD signaling [47,74], JAK2/STAT3 [64] and Wnt/β-catenin [57,64] primarily mediate transdifferentiation and beta-cell neogenesis. Although the studies covered in this review have largely reported on the involvement of single pathways, it would be important to illuminate the other secondary mediators or pathways that may be involved. Indeed, recent studies have shown synergistic associations with small molecules that modulate multiple beta-cell regenerating signaling pathways [158]. Justifiably, any successful endogenous beta-cell regenerative therapies would need to adopt a multifaceted approach. Targeting multiple signaling pathways will elicit an augmented regenerative effect compared to the single pathway effects.

In the context of T2D, beta-cell dysfunction, de-differentiation and apoptosis are resultant of the prevailing diabetic environment that is defined by chronic hyperglycemia, lipotoxicity, oxidative and ER stress and inflammation [198,199,200,201,202,203]. Consequently, any clinically meaningful beta-cell regenerative therapy would need to mitigate the metabolic aberrations that underlie the pathogenesis of diabetes and lead to beta-cell dysfunction and death. This would suggest that beta-cell regeneration would only be meaningful in concert with such other complementary therapeutics. Moreover, an overt diabetes diagnosis occurs when there is already a significant depletion of beta-cell mass. Potentially, therefore, perhaps early diagnosis and treatment would be complementary to any beta-cell regenerative approaches, especially where the target is to stimulate the proliferation of remnant beta cells. Moreover, neogenesis has been noted to be the predominant regenerative mechanism in humans with impaired glucose tolerance or in newly diagnosed T2D [32].

Moreover, not many studies provide the actual quantitative replicative capacity or proliferative rates in cases where the regenerative mechanism is via beta-cell replication. Perhaps it would be useful to have a standardized way to measure these effects that allow us to translate preclinical beta-cell recovery rates to the clinical setting. As such, a particular threshold of recovery/regeneration would directly translate to a particular regenerative rate in human beta cells/or human endocrine cells, as the case may be. Also, the fact that most of the referenced studies have used preclinical models suggests that, although promising, and a lot of these regenerative mechanisms and their mediators are much better understood currently, there still remains a lot of work to be done in determining which plant extracts/compounds/combinations of compounds might offer the best solution for human beta-cell regenerative therapy for T2D. Moreso, given that the regenerative rates differ between rodents, which are primarily used in preclinical studies and human islet cells [18,204,205], it would be important to evaluate the regenerative effects of these phytocompounds in human islets and in the clinical setup. Additionally, more research would be needed to determine whether there exist any indiscriminate effects and hence, what targeting approaches would be applicable to eliminate the non-target effects and eliminate risks for neoplasm formation and tumorigenesis. Also, it is still unclear whether these phytocompound-mediated therapies would need to be administered chronically and, subsequently, what dose would be required to promote adequate beta-cell mass for normoglycemia to be maintained. Perhaps, more long-term studies would address these queries and further establish the stability of the regenerated islets, including their functional and morphological integrity.

## Figures and Tables

**Table 1:** Plant extracts and compounds with pancreatic beta-cell regenerative activity. Summary of studies related to the beta-cell proliferation activities of plant extracts and compounds.

	Plant Source/ Compound	Plant Extract or Compound	Model(s)	Effective Concentration/Dose	Bioactivity	Pathway	References
1.	*Agaricus bisporus*	*Agaricus bisporus* lectin	70% pancreatectomized 8-week-old male C57BL/6J mice	10 mg/kg for 14 days	↑ proliferation of beta cells and duct cells ↑ beta-cell mass ↑ Cdk4 mRNA and protein ↑ cyclin D1 and D2 ↑ Cdk4 activity ↑ Rb phosphorylation ↑ Pdx-1 and Ngn3 mRNA expression	Not indicated	[55]
2.	*Andrographis paniculata*	Andrographolide	PANC-1 cells cultured	1.25, 2.5, 5 μM for 96 h	↑ differentiation of PANC-1 cells into insulin-producing cells Pdx-1 mRNA and protein levels	Not indicated	[56]
			8-week-old 150mg/kg STZ-diabetic male Kunming mice	Transplanted with 200 islets from normal mice or 500 differentiated islet-like cell clusters into the renal capsular for 5 days Or treated with 50 mg/kg daily for 40 days	↑ Pdx-1 mRNA and protein levels ↑ insulin^+^ and Pdx-1^+^ islets Restored islet morphology Pathway not indicated	Not indicated	[56]
3.	*Aralia taibaiensis*	Chikusetsu saponin IVa	βTC3 cell line exposed to high glucose (33.3 mM)	40 µM for 0, 6, 12, 24, 36 or 48 h	↑ cell viability and proliferation ↑ TCF7L2, Wnt3a mRNA and protein expression ↑ nuclear β-catenin levels ↑ p-GSK-3β ↑ c-Myc, cyclin D1, skp2 protein levels ↓ p53, p21 and p27 protein levels	Wnt/β-catenin/TCF7L2	[57]
			Pancreatic islet cells from male Sprague Dawley rats exposed to high glucose	40 µM for 24 h	↑ Wnt3a mRNA and protein ↑ nuclear β-catenin ↑ p-GSK-3β ↑ TCF7L2, c-Myc, cyclin D1, skp2 protein levels ↓ p53, p21 and p27	Wnt/β-catenin/TCF7L2	[57]
			β-catenin gene knockout mice (β-catenin−/−) 4-week-old male mice, HFD-fed for 8 weeks then given 50 mg/kg bwt STZ	120 mg/kg for 30 days	↑ Wnt3a mRNA and protein ↑ nuclear β-catenin levels ↑ p-GSK-3β ↑ TCF7L2, c-Myc, cyclin D1, skp2 protein levels ↓ p53, p21 and p27	Wnt/β-catenin/TCF7L2	[57]
4.	*Ervatamia microphylla*	Conophylline	AR42J-B13 cells	0.1 μg/mL for 9 or 72 h	Differentiation of AR42J cells into insulin-expressing cells ↑ Ngn3, NeuroD and Nkx2.2 mRNA expression ↑ phosphorylation of p38	Not indicated	[58]
			14.5-day-old embryonic pancreata	0.1 µg/mL for 72 h or 0.1 µg/mL every 2 days for 10 days	↑ number of islet-like cell clusters ↑ number of insulin^+^ cells ↑ β-cell differentiation ↑ insulin^+^/Pdx-1^+^ cells around duct-like structures ↑ insulin^+^/Pdx-1^+^ area	Not indicated	[59]
			Endocrine cells from neonatal porcine pancreas Islet-like cell clusters from the pancreases of new-born pigs	0.1 µg/mL conophylline alone or combined with 10 mM nicotinamide treated for 1, 3, 5 or 6 weeks	↑ number of insulin-producing cells ↑ differentiation of ICC into functional glucose-responsive cells ↑ mRNA levels of Ngn3, Pdx-1, NeuroD in islet-like cell clusters	Not indicated	[60]
			STZ-induced one-day-old male neonatal Wistar rats	5 µg/g subcutaneously on days 1, 3, 5 and 7	↑ relative area for Pdx-1/insulin-positive cells ↑ Pdx-1^+^ ductal cells ↑ number of islet-like cell clusters ↑ β-cell mass	Not indicated	[59]
			STZ-induced one-day-old male neonatal Wistar rats	2 μg/g (i.p) conophylline every other day until day 7 or 200 pmol/g betacellulin daily for 7 days with 2 μg/g conophylline every other day for 7 days	↑ β-cell mass ↑ number and size of islet-like cell clusters ↑ number of insulin^+^/BrdU^+^ cells ↑ Pdx-1^+/^CK-19^+^ ductal cells	Not indicated	[61]
5.		Epigallocatechin gallate, Rutin	Endocrine cells from neonatal porcine pancreas Islet-like cell clusters from the pancreases of new-born pigs	0.1 µg/mL conophylline alone or combined with 10 mM nicotinamide treated for 1, 3, 5 or 6 weeks	↑ number of insulin-producing cells ↑ differentiation of ICC into functional glucose-responsive cells ↑ mRNA levels of Ngn3, Pdx-1, NeuroD in islet-like cell clusters	Not indicated	[60]
		Epigallocatechin gallate (EGCG)	7-week-old male *db*/*db* mice	10 g/kg of diet (EGCG 1% [*w*/*w*]) for 10 weeks	↑ number of islets, islet size, endocrine area ↓ Cdkn1a and Ppp1r15a	Not indicated	[62]
6.		FTY720 (Fingolimod)	Five-week-old female *db*/*db mice* (BKS.Cg-*m+*/*+Leprdb*)	10 mg/kg of FTY720 daily via oral gavage for 6 weeks	↑ β-cell mass, islet area ↑ proliferation of beta and duct cells ↑ differentiation of ductal cells to insulin^+^ cells ↑ expression of Pdx-1, cyclins D1, D2 and D3 ↓ p57^KIP2^	PI3K/Akt pathway via Sphingosine 1-phosphate receptors	[63]
7.	*Gardenia jasminoides* Ellis	Geniposide	MIN6 cells exposed to high glucose (33.3 mM) or a mixture of IL-1β plus IFN-γ	20 μM for 3 days	↑ p-Akt, p-GSK-3β, nuclear β-catenin and PKA C-α protein expression	Wnt/β-catenin/TCF7L2	[64]
			Primary islets from male C57BL/6J mice exposed to high glucose (33.3 mM) or a mixture of IL-1β plus IFN-γ and exocrine cells from male C57BL/6J mice	20 µM for 3 days for islets 20 µM for 4 days for exocrine cells	↑ β-cell proliferation ↑ mRNA expression of Pdx-1, TCF7L2, cyclin D1 ↑ protein expression of TCF7L2 and p-Akt Islet-like clusters from ductal cells Pdx-1^+^, insulin^+^, MafA^+^, ductal cells ↑ Pdx-1 and insulin mRNA in exocrine cells	Wnt/β-catenin/TCF7L2 and JAK2/STAT3 signaling	[64]
			4-week-old male C57BL/6J mice fed an HFD for 12 weeks 4-week-old male C57Bl/KsJ (BKS) mice and BKS.Cg-Dock7m+/+Leprdb/JNju (*db*/*db*)	100 mg/kg for 4 weeks 100 mg/kg for 35 days	↑ β-cell mass and proliferation ↑ Pdx-1^+^ duct cells ↑ Ngn3^+^ duct cells ↑ islet-like cell clusters within ducts ↑ TCF7L2^+^ beta and duct cells	Wnt/β-catenin/TCF7L2 and JAK2/STAT3 signaling	[64]
8.	Various leguminous plants	Genistein	INS-1 cells	1 uM genistein for 15 min or 24 h	↑ β-cell proliferation ↑ cyclin D expression ↑ ERK1/2 phosphorylation	cAMP/PKA and MEK/ERK signaling pathways	[65]
			Human islet beta cells	1 µM and 5 µM genistein for 24 h	↑ β-cell proliferation ↑ cyclin D expression ↑ cAMP levels and activation of PKA in human islets	cAMP/PKA and MEK/ERK signaling pathways	[65]
			High-fat diet + STZ-diabetic 4-week-old male C57BL/6J mice	0.25 g/kg in the diet for 28 days	↑ β-cell proliferation	cAMP/PKA and MEK/ERK signaling pathways	[65]
9.	*Glycine max* (Soybean)	*G. max* leaves compounds (Kaempferol and pheophorbide a)	MIN6 pancreatic β-cells	50 uM or 100 uM kaempferol for 48 h 0.1 or 1 μM pheophorbide a	Kaempferol - ↑mRNA levels of IRS2 ↑ Akt ↑ FoxO1 phosphorylation ↑ gene expression of Pdx-1, Ngn3 and Pax4 ↑ β-cell proliferation Pheophorbide a - ↑mRNA levels of IRS1 ↑ Ngn3, Pax4 and PKA gene expression ↑ induction of Akt and PKA phosphorylation ↑ β-cell proliferation	For kaempferol -IRS2/PI3K/Akt signaling via FoxO1 Pheophorbide a via IRS1/PI3K/Akt/PKA signaling	[66]
		S-equol	INS-1 cells exposed to 1 mMol/L streptozotocin	10 μMol/L for 24 h	↑ cell proliferation	cAMP/PKA signaling	[67]
			STZ-induced 5-week-old male ICR mice	20 mg/kg via oral gavage twice daily for 7 days	↑ β-cell proliferation	cAMP/PKA signaling	[67]
		Ethyl acetate extract of *G. max* leaves or pinitol	High-fat diet-fed 4-week-old male C57BL/6J mice	0.56% extract or 0.15% pinitol in the diet for 12 weeks	For extract- ↑ islet size, Ngn3 and MafA mRNA ↓ FoxO1 mRNA For pinitol- ↑ islet size, ↑ Ngn3, Pax4, MafA and IRS1 mRNA	Insulin signaling pathway	[68]
		Ethanol extract of *G. max* leaves	5-week-old male C57BLKS/J lar-*Leprdb*/*Leprdb* (*db*/*db*) and C57BLKS/J lar-m+/*Leprdb* (*db*/+) mice	1% extract for 8 weeks	↑ gene expression of IRS1, IRS2, Pdx-1, Ngn3, and Pax4 ↓ FoxO1 gene expression ↑ FoxO1 phosphorylation	IRS2/Akt signaling via FoxO1	[66]
10.	Ginseng	Ginsenoside Rh2 (GS-Rh2)	70% pancreatectomized- 3-month-old male C57BL/6J mice	1 mg/kg (i.p) for 14 days	↑ beta-cell proliferation and mass ↑ p-Akt, p-FoxO1, Pdx-1, cyclin D1, cyclin D2 and Cdk4 protein ↑ Pdx-1, cyclin D1, cyclin D2 and Cdk4 mRNA ↑ Cdk4 activity	Akt/FoxO1/Pdx1 signaling	[69]
	Ginseng	Ethanol extract of *G. radix*	Isolated pancreatic islets from male rats incubated for 24h in high glucose (20 mM)	50 μg/mL for 8 h	↑ IRS2 mRNA ↑ Pdx-1 mRNA	IRS2/IGF-1/Pdx-1 signaling	[70]
	*Panax ginseng*	Ethanol extracts of ripe and unripe ginseng berries	INS-1 rat insulinoma cells STZ-induced -8-week-old male C57BL/6 mice	5, 10, and 20 µg/mL extract for 24 h 100 or 200 mg/kg extract via oral intubation daily for 10 weeks	↑ β-cell numbers ↑ cyclin D2, Pdx-1, and IRS2 mRNA ↑ serum insulin	Not indicated	[71]
11.	*Gymnema sylvestre*	*Gymnemic acid from G. sylvestre* leaf	2-month-old STZ-induced male Wistar rats RIN5-F beta cells exposed to 25 mM high glucose for 48 h	150 mg/kg b.w; via oral gavage for 30 days. 1 µM for 24 h	↑ Pdx-1, Ngn3, MafA and NeuroD1 mRNA and protein ↑ E-cadherin, β-catenin, PI3K, AKT, pAKT, Cyclin D1 and CDK4 ↓ FoxO1, GSK-3β and p21cip1 ↑ nuclear localization of Pdx-1	PI3K/Akt signaling	[72]
12.	*Rhodiola rosea*	Salidroside	Mouse islets from -10-week-old *db*/*db* and -10-week-old C57BL/6 mice exposed to diabetic 33.3 mM glucose, the mixture of 2 ng/mL IL-1 β +1000 U/mL IFN-γ, 0.5 mM palmitic acid or 200 µM H_2_O_2_	50 µM for 3 days	↑ β-cell proliferation ↑ nuclear Pdx-1 ↓ nuclear FoxO1	Akt/FoxO1 signaling	[73]
			4-week-old male C57BL/6 mice (HFD-fed) C57Bl/KsJ (BKS) mice BKS.Cg-Dock7m+/+Leprdb/J (*db*/*db*) mice	100 mg/kg/day via oral gavage for 5 weeks	↑ β-cell mass and proliferation	Akt/FoxO1 signaling	[73]
13.	*Enicostemma littorale*	Swertisin	NIH3T3 cells	15 µg/mL for 8 days	Initial ↑ gene and protein expression of nestin, Pdx-1, Ngn3, Pax4, Nkx6.1 and Reg-1 later ↓ expression of stem cell markers nestin, vimentin and SMA↑ islet cell differentiation ↓ Smad7 expression ↑ Smad2 expression	SMAD signaling	[74]
			PANC-1 cells	15 µg/mL for 8 days	↑ differentiation into islet-like cell clusters ↑ gene expression of nestin, p38 phosphorylation, unchanged Pdx-1 expression early ↑ and late ↓ Ngn3 expression	MEPK-TKK pathway via p38 phosphorylation SMAD signaling	[47]
			Mouse intra-islet progenitor cells	15 μg/mL for eight days	↑ islet differentiation of mouse intra-islet pancreatic progenitor cells into beta cells showing ↑ Ngn3 ↓ Erk1/2 levels ↑ Pax4 expression ↑ n-cadherin ↓ SMAD2/3/7	MEPK-TKK pathway via p38 phosphorylation SMAD signaling	[47]
			70% pancreatectomized 3–4-week-old male Balb/c mice	Single injection of swertisin-dose not given	↓ Pdx-1 ↑ nestin expression ↑ Ngn3 expression ↓ Smad 7 expression ↑ Smad-2/3 phosphorylation ↑ activation of MAP kinase ↑ differentiation of progenitor cells within the acinar and islet tissues	MEPK-TKK pathway SMAD signaling	[47]
			STZ-diabetic 6–8-week-old female Balb/c mice	2.5 mg/kg for 17 days	↑ nestin, Pdx-1, Ngn3, MafA and Nkx6.1 protein expression	Not indicated	[75]
14.	*Mangifera indica*	Mangiferin	Islet cells from male adult (age 3 months) and aged (age 12 months) mice	Cells incubated with mangiferin for 24 h (concentration not given)	↑ Cdk4 activity ↑ inhibition of p16 ↑ expression and phosphorylation of STAT3	STAT3 signaling	[76]
			70% pancreatectomized 8-week-old male C57BL/6J mice	30 mg/kg or 90 mg/kg for 14 days	↑ absolute β-cell mass ↑ beta and duct cell proliferation ↑ cyclin D1, cyclin D2 and Cdk4 mRNA and protein expression ↑ Cdk4 activity ↑ Rb phosphorylation ↓ p27 mRNA and protein levels ↑ FoxO1, Pdx-1 and Ngn3 mRNA and protein expression Not indicated		[77]
			70% pancreatectomized 12-month-old C57BL/6J mice	90 mg/kg (i.p.) for 28 days	↑ proliferation of the islet cells ↑ β-cell volume and mass ↑ transcription and translation of Pdx-1, cyclin D1, D2 and Cdk4 ↓ expression of p16^INK4a^ and p27^Kip1^ ↑ expression and phosphorylation of STAT3 ↓ phosphorylated Rb ↑ Cdk4 activity	STAT3 signaling	[76]
15.	*Oreocnide integrifolia*	Flavonoid-rich fraction of *O. integrifolia*	70% pancreatectomized 7–8-week-old female Balb/c mice	250 mg/kg for 7, 14, and 21 days	↑ Pdx-1/insulin co-expressing cells ↑ number of neogenic islet nodes ↑ β-cell density ↑ proliferation of ductal precursor cells ↑ Ngn3, Pdx-1 and Reg- 3α/γ mRNA CK-19/insulin co-expression in ducts	Not indicated	[78]
16.	*Radix puerariae*	Puerarin	Pancreatic ductal cells from 5-week-old male C57BL/6 mice fed a high-fat diet	50 µM for 3 days	↑ Ngn3, Pdx-1, and insulin expression ↑ GLP-1R expression, activation of β-catenin, JAK2 and STAT3 in ductal cells ↑ Pdx-1^+^/CK19^+^ staining ↑ ICC formation	GLP-1R/Wnt/STAT signaling	[79]
			High-fat diet-fed 5-week-old male C57BL/6 mice	150 or 300 mg/kg per day for 10 or 20 days via oral gavage	↑ β-cell proliferation ↑ Ngn3^+^ and Pdx-1^+^ duct cells ↑ islet-like cell clusters next duct cells	GLP-1R/Wnt/STAT signaling	[79]
17.	Commercially sourced	Quercetin	7-week-old STZ- induced male BALB/c mice	0.1 or 0.5% (*w*/*w*) quercetin in the diet for 2 weeks	↓ Cdkn1a gene expression	Not indicated	[80]
18.	*Rosa canina*	Oligosaccharide isolated from *Rosa canina* (ripe fruits)	STZ-induced 8-week-old male Wistar rats	8–40 mg/kg of oligosaccharide twice a day for 21 days via oral gavage	↑ β-cell mass ↑ Ngn3 and Nkx6.1 expression	Not indicated	[81]
19.	*Silybum marianum*	Silymarin	Alloxan-induced male Wistar rats	200 mg/kg for 9 weeks	Normalized Pdx-1 protein and pancreas histology	Not indicated	[42]
			70% pancreatectomized male Wistar rats	200 mg/kg (p.o.) for 3, 7, 14, 21, 42 and 63 days	↑ Pdx-1 gene and protein expression ↑ β-cell proliferation	Not indicated	[82]
20.	*Tinospora cordifolia*	*T. cordifolia* aqueous extract	PANC-1 cells	15 μg/mL	↑ Pdx-1 mRNA expression ↓ Carbonic anhydrase 9 mRNA expression	Not indicated	[83]

Note: ↑ depicts an increase in mRNA or protein expression or activity, ↓ depicts a decrease in mRNA or protein expression or activity

## Data Availability

Not applicable.

## References

[1] Whiting D.R., Guariguata L., Weil C., Shaw J. (2011). IDF Diabetes Atlas: Global Estimates of the Prevalence of Diabetes for 2011 and 2030. Diabetes Res. Clin. Pract..

[2] Ogurtsova K., Da J.D., Fernandes R., Huang Y., Linnenkamp U., Guariguata L., Cho N.H., Cavan D., Shaw J.E., Makaroff L.E. (2017). IDF Diabetes Atlas: Global Estimates for the Prevalence of Diabetes for 2015 and 2040. Diabetes Res. Clin. Pract..

[3] Saeedi P., Petersohn I., Salpea P., Malanda B., Karuranga S., Unwin N., Colagiuri S., Guariguata L., Motala A.A., Ogurtsova K. (2019). Global and Regional Diabetes Prevalence Estimates for 2019 and Projections for 2030 and 2045: Results from the International Diabetes Federation Diabetes Atlas, 9th Edition. Diabetes Res. Clin. Pract..

[4] Sinclair A., Saeedi P., Kaundal A., Karuranga S., Malanda B., Williams R. (2020). Diabetes and Global Ageing among 65–99-Year-Old Adults: Findings from the International Diabetes Federation Diabetes Atlas, 9th Edition. Diabetes Res. Clin. Pract..

[5] Saeedi P., Salpea P., Karuranga S., Petersohn I., Malanda B., Gregg E.W., Unwin N., Wild S.H., Williams R. (2020). Mortality Attributable to Diabetes in 20–79 Years Old Adults, 2019 Estimates: Results from the International Diabetes Federation Diabetes Atlas, 9th Edition. Diabetes Res. Clin. Pract..

[6] Aronoff S.L., Berkowitz K., Shreiner B., Want L. (2004). Glucose Metabolism and Regulation: Beyond Insulin and Glucagon. Diabetes Spectr..

[7] Krentz A.J., Bailey C.J. (2005). Oral Antidiabetic Agents: Current Role in Type 2 Diabetes Mellitus. Drugs.

[8] Litwak L., Goh S.-Y., Hussein Z., Malek R., Prusty V., Khamseh M.E. (2013). Prevalence of Diabetes Complications in People with Type 2 Diabetes Mellitus and Its Association with Baseline Characteristics in the Multinational A 1 Chieve Study. Diabetol. Metab. Syndr..

[9] Chala T.S., Ali G.Y. (2016). Recent Advance in Diabetes Therapy: Pancreatic Beta Cell Regeneration Approaches. Diabetes Manag..

[10] Domínguez-Bendala J., Inverardi L., Ricordi C. (2012). Regeneration of Pancreatic Beta-Cell Mass for the Treatment of Diabetes. Expert Opin. Biol. Ther..

[11] Aguayo-Mazzucato C., Bonner-Weir S. (2018). Pancreatic β Cell Regeneration as a Possible Therapy for Diabetes. Cell Metab..

[12] Hosseini A., Shafiee-Nick R., Ghorbani A. (2015). Pancreatic Beta Cell Protection/Regeneration with Phytotherapy. Braz. J. Pharm. Sci..

[13] Cai H., Cheng R., Yuan R., Zhu X., Ao P. (2018). Systems Biology Theory Clarification of a Controversy in Pancreatic Beta Cell Regeneration. bioRxiv.

[14] Zhong F., Jiang Y. (2019). Endogenous Pancreatic β Cell Regeneration: A Potential Strategy for the Recovery of β Cell Deficiency in Diabetes. Front. Endocrinol..

[15] Banerjee M., Kanitkar M., Bhonde R.R. (2005). Approaches Towards Endogenous Pancreatic Regeneration. Rev. Diabet. Stud..

[16] Dor Y., Brown J., Martinez O.I., Melton D.A. (2004). Adult Pancreatic β-Cells Are Formed by Self-Duplication Rather than Stem-Cell Differentiation. Nature.

[17] Bentz J., O’Connor M.P., Bednarczyk D., Coleman J., Lee C., Palm J., Pak Y.A., Perloff E.S., Reyner E., Balimane P. (2013). Variability in P-Glycoprotein Inhibitory Potency (IC 50) Using Various in Vitro Experimental Systems: Implications for Universal Digoxin Drug-Drug Interaction Risk Assessment Decision Criteria. Drug Metab. Dispos..

[18] Wang P., Fiaschi-Taesch N.M., Vasavada R.C., Scott D.K., García-Ocaña A., Stewart A.F. (2015). Diabetes Mellitus—Advances and Challenges in Human β-Cell Proliferation. Nat. Rev. Endocrinol..

[19] Meier J., Butler A., Saisho Y., Monchamp T., Galasso R., Bhushan A., Rizza R.A., Butler P.C. (2008). β-Cell Replication Is the Primary Mechanism Subserving the Postnatal Expansion of β-Cell Mass in Humans. Diabetes.

[20] Peshavaria M., Larmie B.L., Lausier J., Satish B., Habibovic A., Roskens V., LaRock K., Everill B., Leahy J.L., Jetton T.L. (2006). Regulation of Pancreatic β-Cell Regeneration in the Normoglycemic 60% Partial-Pancreatectomy Mouse. Diabetes.

[21] Rieck S., Kaestner K.H. (2010). Expansion of β-Cell Mass in Response to Pregnancy. Trends Endocrinol. Metab..

[22] Li W.-C., Chen C.-Y., Chien H.-Y., Bonner-Weir S., Hardikar A.A. (2016). Pancreatic Regeneration After Partial Pancreatectomy in Rodents. Pancreatic Islet Biology, Stem Cell Biology and Regenerative Medicine.

[23] Bouwens L., Rooman I. (2005). Regulation of Pancreatic Beta-Cell Mass. Physiol. Rev..

[24] Cinti F., Bouchi R., Kim-Muller J.Y., Ohmura Y., Sandoval P.R., Masini M., Marselli L., Suleiman M., Ratner L.E., Marchetti P. (2016). Evidence of β-Cell Dedifferentiation in Human Type 2 Diabetes. J. Clin. Endocrinol. Metab..

[25] Guo S. (2013). Molecular Basis of Insulin Resistance: The Role of IRS and Foxo1 in the Control of Diabetes Mellitus and Its Complications. Drug Discov. Today Dis. Mech..

[26] Hunter C.S., Stein R.W. (2017). Evidence for Loss in Identity, De-Differentiation, and Trans-Differentiation of Islet β-Cells in Type 2 Diabetes. Front. Genet..

[27] Sheng C., Li F., Lin Z., Zhang M., Yang P., Bu L., Sheng H., Li H., Qu S. (2016). Reversibility of β-Cell-Specific Transcript Factors Expression by Long-Term Caloric Restriction in Db/Db Mouse. J. Diabetes Res..

[28] Guo S., Dai C., Guo M., Taylor B., Harmon J.S., Sander M., Robertson R.P., Powers A.C., Stein R. (2013). Inactivation of Specific β Cell Transcription Factors in Type 2 Diabetes. J. Clin. Investig..

[29] Gribben C., Lambert C., Messal H.A., Hubber E.L., Rackham C., Evans I., Heimberg H., Jones P., Sancho R., Behrens A. (2021). Ductal Ngn3-Expressing Progenitors Contribute to Adult β Cell Neogenesis in the Pancreas. Cell Stem Cell.

[30] Butler A.E., Cao-Minh L., R (2010). Galasso; Rizza, R.A.; Corradin, A.; Cobelli, C.; Butler, P.C. Adaptive Changes in Pancreatic Beta Cell Fractional Area and Beta Cell Turnover in Human Pregnancy. Diabetologia.

[31] Mezza T., Muscogiuri G., Sorice G.P., Clemente G., Hu J., Pontecorvi A., Holst J.J., Giaccari A., Kulkarni R.N. (2014). Insulin Resistance Alters Islet Morphology in Nondiabetic Humans. Diabetes.

[32] Yoneda S., Uno S., Iwahashi H., Fujita Y., Yoshikawa A., Kozawa J., Okita K., Takiuchi D., Eguchi H., Nagano H. (2013). Predominance of β-Cell Neogenesis Rather than Replication in Humans with an Impaired Glucose Tolerance and Newly Diagnosed Diabetes. J. Clin. Endocrinol. Metab..

[33] Qadir M.M.F., Álvarez-Cubela S., Klein D., van Dijk J., Muñiz-Anquela R., Moreno-Hernández Y.B., Lanzoni G., Sadiq S., Navarro-Rubio B., García M.T. (2020). Single-Cell Resolution Analysis of the Human Pancreatic Ductal Progenitor Cell Niche. Proc. Natl. Acad. Sci. USA.

[34] Huising M.O., Lee S., van der Meulen T. (2018). Evidence for a Neogenic Niche at the Periphery of Pancreatic Islets. BioEssays.

[35] Wang D., Wang J., Bai L., Pan H., Feng H., Clevers H., Zeng Y.A. (2020). Long-Term Expansion of Pancreatic Islet Organoids from Resident Procr+ Progenitors. Cell.

[36] Wang W., Liu C., Jimenez-Gonzalez M., Song W.-J., Hussain M.A. (2017). The Undoing and Redoing of the Diabetic β-Cell. J. Diabetes Complicat..

[37] Brereton M.F., Iberl M., Shimomura K., Zhang Q., Adriaenssens A.E., Proks P., Spiliotis I.I., Dace W., Mattis K.K., Ramracheya R. (2014). Reversible Changes in Pancreatic Islet Structure and Function Produced by Elevated Blood Glucose. Nat. Commun..

[38] Wang Z., York N.W., Nichols C.G., Remedi M.S. (2014). Pancreatic β Cell Dedifferentiation in Diabetes and Redifferentiation Following Insulin Therapy. Cell Metab..

[39] Agwaya M., Nandutu A., Vuzi P. (2016). Protective Effects of Zanthoxylum Chalybeum in Diabetes-Induced Myocardial Dysfunction in Rats. Eur. J. Med. Plants.

[40] Bahar E., Akter K.-M., Lee G.-H., Lee H.-Y., Rashid H.-O., Choi M.-K., Bhattarai K.R., Hossain M.M.M., Ara J., Mazumder K. (2017). β-Cell Protection and Antidiabetic Activities of *Crassocephalum crepidioides* (Asteraceae) Benth. S. Moore Extract against Alloxan-Induced Oxidative Stress via Regulation of Apoptosis and Reactive Oxygen Species (ROS). BMC Complement. Altern. Med..

[41] Ramadan B.K., Schaalan M.F., Tolba A.M. (2017). Hypoglycemic and Pancreatic Protective Effects of Portulaca Oleracea Extract in Alloxan Induced Diabetic Rats. BMC Complement. Altern. Med..

[42] Soto C., Mena R., Luna J., Cerbón M., Larrieta E., Vital P., Uría E., Sánchez M., Recoba R., Barrón H. (2004). Silymarin Induces Recovery of Pancreatic Function after Alloxan Damage in Rats. Life Sci..

[43] Abdel Aziz M.T., El-Asmar M.F., Rezq A.M., Mahfouz S.M., Wassef M.A., Fouad H.H., Ahmed H.H., Taha F.M. (2013). The Effect of a Novel Curcumin Derivative on Pancreatic Islet Regeneration in Experimental Type-1 Diabetes in Rats (Long Term Study). Diabetol. Metab. Syndr..

[44] Rajalakshmi M., Anita R. (2016). β-Cell Regenerative Efficacy of a Polysaccharide Isolated from Methanolic Extract of *Tinospora cordifolia* Stem on Streptozotocin -Induced Diabetic Wistar Rats. Chem. Biol. Interact..

[45] Ranjbari A., Azarbayjani M.A., Yusof A., Mokhtar A.H., Akbarzadeh S., Ibrahim M.Y., Tarverdizadeh B., Farzadinia P., Hajiaghaee R., Dehghan F. (2016). In Vivo and in Vitro Evaluation of the Effects of Urtica Dioica and Swimming Activity on Diabetic Factors and Pancreatic Beta Cells. BMC Complement. Altern. Med..

[46] Saleh F.A., El-Darra N., Raafat K. (2017). Hypoglycemic Effects of *Prunus cerasus* L. Pulp and Seed Extracts on Alloxan-Induced Diabetic Mice with Histopathological Evaluation. Biomed. Pharmacother..

[47] Dadheech N., Srivastava A., Paranjape N., Gupta S., Dave A., Shah G.M., Bhonde R.R., Gupta S. (2015). Swertisin an Anti-Diabetic Compound Facilitate Islet Neogenesis from Pancreatic Stem/Progenitor Cells via p-38 MAP Kinase-SMAD Pathway: An In-Vitro and In-Vivo Study. PLoS ONE.

[48] Mathijs I., Da Cunha D.A., Himpe E., Ladriere L., Chellan N., Roux C.R., Joubert E., Muller C., Cnop M., Louw J. (2014). Phenylpropenoic Acid Glucoside Augments Pancreatic Beta Cell Mass in High-Fat Diet-Fed Mice and Protects Beta Cells from ER Stress-Induced Apoptosis. Mol. Nutr. Food Res..

[49] Oh Y.S. (2015). Plant-Derived Compounds Targeting Pancreatic Beta Cells for the Treatment of Diabetes. Evid.-Based Complement. Altern. Med..

[50] Ghorbani A., Rashidi R., Shafiee-Nick R. (2019). Flavonoids for Preserving Pancreatic Beta Cell Survival and Function: A Mechanistic Review. Biomed. Pharmacother..

[51] Oliaee D., Niazkar H.R., Abbasnezhad A., Ghorbani M., Alavi Shahri P.S., Saghaee Shahri S., Ghanaiyan K. (2020). The Effects of Medicinal Plants on Pancreatic Beta Cells in Diabetes: A Systematic Review of Iranians’ Contributions. Rev. Clin. Med..

[52] Apaya M.K., Kuo T.F., Yang M.T., Yang G., Hsiao C.L., Chang S.-B., Lin Y., Yang W.C. (2020). Phytochemicals as Modulators of β-Cells and Immunity for the Therapy of Type 1 Diabetes: Recent Discoveries in Pharmacological Mechanisms and Clinical Potential. Pharmacol. Res..

[53] Attanayake A.P., Jayatilaka K.A.P.W., Mudduwa L.K.B., Pathirana C. (2018). β-Cell Regenerative Potential of Selected Herbal Extracts in Alloxan Induced Diabetic Rats. Curr. Drug Discov. Technol..

[54] Tiwari A.K., Rao J.M. (2002). Diabetes Mellitus and Multiple Therapeutic Approaches of Phytochemicals: Present Status and Future Prospects. Curr. Sci..

[55] Wang Y., Liu Y., Wang H., Li C., Qi P., Bao J. (2012). Agaricus Bisporus Lectins Mediates Islet β-Cell Proliferation through Regulation of Cell Cycle Proteins. Exp. Biol. Med..

[56] Zhang S., Huang F., Tian W., Lai J., Qian L., Hong W., Chen H., Li L. (2020). Andrographolide Promotes Pancreatic Duct Cells Differentiation into Insulin-Producing Cells by Targeting PDX-1. Biochem. Pharmacol..

[57] Cui J., Duan J., Chu J., Guo C., Xi M., Li Y., Weng Y., Wei G., Yin Y., Wen A. (2020). Chikusetsu Saponin IVa Protects Pancreatic β Cell against Intermittent High Glucose-Induced Injury by Activating Wnt/β-Catenin/TCF7L2 Pathway. Aging.

[58] Umezawa K., Hiroki A., Kawakami M., Naka H., Takei I., Ogata T., Kojima I., Koyano T., Kowithayakorn T., Pang H.S. (2003). Induction of Insulin Production in Rat Pancreatic Acinar Carcinoma Cells by Conophylline. Biomed. Pharmacother..

[59] Ogata T., Li L., Yamada S., Yamamoto Y., Tanaka Y., Takei I., Umezawa K., Kojima I. (2004). Promotion of Beta-Cell Differentiation by Conophylline in Fetal and Neonatal Rat Pancreas. Diabetes.

[60] Kawakami M., Hirayama A., Tsuchiya K., Ohgawara H., Nakamura M., Umezawa K. (2010). Promotion of β-Cell Differentiation by the Alkaloid Conophylline in Porcine Pancreatic Endocrine Cells. Biomed. Pharmacother..

[61] Kodera T., Yamada S., Yamamoto Y., Hara A., Tanaka Y., Seno M., Umezawa K., Takei I., Kojima I. (2009). Administration of Conophylline and Betacellulin-Delta4 Increases the Beta-Cell Mass in Neonatal Streptozotocin-Treated Rats. Endocr. J..

[62] Ortsäter H., Grankvist N., Wolfram S., Kuehn N., Sjöholm Å. (2012). Diet Supplementation with Green Tea Extract Epigallocatechin Gallate Prevents Progression to Glucose Intolerance in Db/Db Mice. Nutr. Metab..

[63] Zhao Z., Choi J., Zhao C., Ma Z.A. (2012). FTY720 Normalizes Hyperglycemia by Stimulating β-Cell in Vivo Regeneration in Db/Db Mice through Regulation of Cyclin D3 and P57 KIP2. J. Biol. Chem..

[64] Yao D.D., Yang L., Wang Y., Liu C., Wei Y.J., Jia X.B., Yin W., Shu L. (2015). Geniposide Promotes Beta-Cell Regeneration and Survival through Regulating β-Catenin/TCF7L2 Pathway. Cell Death Dis..

[65] Fu Z., Zhang W., Zhen W., Lum H., Nadler J., Bassaganya-Riera J., Jia Z., Wang Y., Misra H., Liu D. (2010). Genistein Induces Pancreatic β-Cell Proliferation through Activation of Multiple Signaling Pathways and Prevents Insulin-Deficient Diabetes in Mice. Endocrinology.

[66] Li H., Ji H.S., Kang J.H., Shin D.H., Park H.Y., Choi M.S., Lee C.H., Lee I.K., Yun B.S., Jeong T.S. (2015). Soy Leaf Extract Containing Kaempferol Glycosides and Pheophorbides Improves Glucose Homeostasis by Enhancing Pancreatic β-Cell Function and Suppressing Hepatic Lipid Accumulation in Db/Db Mice. J. Agric. Food Chem..

[67] Horiuchi H., Usami A., Shirai R., Harada N., Ikushiro S., Sakaki T., Nakano Y., Inui H., Yamaji R. (2017). *S*-Equol Activates CAMP Signaling at the Plasma Membrane of INS-1 Pancreatic β-Cells and Protects against Streptozotocin-Induced Hyperglycemia by Increasing β-Cell Function in Male Mice. J. Nutr..

[68] Kim U.H., Yoon J.H., Li H., Kang J.H., Ji H.S., Park K.H., Shin D.H., Park H.Y., Jeong T.S. (2014). Pterocarpan-Enriched Soy Leaf Extract Ameliorates Insulin Sensitivity and Pancreatic β-Cell Proliferation in Type 2 Diabetic Mice. Molecules.

[69] Wang Y., Wang H., Liu Y., Li C., Qi P., Bao J. (2012). Antihyperglycemic Effect of Ginsenoside Rh2 by Inducing Islet β-Cell Regeneration in Mice. Horm. Metab. Res..

[70] Park S.M., Hong S.M., Sung S.R., Lee J.E., Kwon D.Y. (2008). Extracts of Rehmanniae Radix, Ginseng Radix and Scutellariae Radix Improve Glucose-Stimulated Insulin Secretion and β-Cell Proliferation through IRS2 Induction. Genes Nutr..

[71] Park E.Y., Kim H.J., Kim Y.K., Park S.U., Choi J.E., Cha J.Y., Jun H.S. (2012). Increase in Insulin Secretion Induced by Panax Ginseng Berry Extracts Contributes to the Amelioration of Hyperglycemia in Streptozotocininduced Diabetic Mice. J. Ginseng Res..

[72] Kannan P., Raghunathan M., Mohan T., Palanivelu S., Periandavan K. (2022). Gymnemic Acid Ameliorates Pancreatic β-Cell Dysfunction by Modulating Pdx1 Expression: A Possible Strategy for β-Cell Regeneration. Tissue Eng. Regen. Med..

[73] Ju L., Wen X., Wang C., Wei Y., Peng Y., Ding Y., Feng L., Shu L. (2017). Salidroside, a Natural Antioxidant, Improves β-Cell Survival and Function via Activating AMPK Pathway. Front. Pharmacol..

[74] Dadheech N., Soni S., Srivastava A., Dadheech S., Gupta S., Gopurappilly R., Bhonde R.R., Gupta S. (2013). A Small Molecule Swertisin from Enicostemma Littorale Differentiates NIH3T3 Cells into Islet-like Clusters and Restores Normoglycemia upon Transplantation in Diabetic Balb/c Mice. Evid.-Based Complement. Altern. Med..

[75] Srivastava A., Dadheech N., Vakani M., Gupta S. (2018). Swertisin Ameliorates Diabetes by Triggering Pancreatic Progenitors for Islet Neogenesis in Streptozotocin Treated BALB/c Mice. Biomed. Pharmacother..

[76] Wang H., He X., Lei T., Liu Y., Huai G., Sun M., Deng S., Yang H., Tong R., Wang Y. (2018). Mangiferin Induces Islet Regeneration in Aged Mice through Regulating P16INK4a. Int. J. Mol. Med..

[77] Wang H.L., Li C.Y., Zhang B., Liu Y.D., Lu B.M., Shi Z., An N., Zhao L.K., Zhang J.J., Bao J.K. (2014). Mangiferin Facilitates Islet Regeneration and β-Cell Proliferation through Upregulation of Cell Cycle and β-Cell Regeneration Regulators. Int. J. Mol. Sci..

[78] Ansarullah, Bharucha B., Umarani M., Dwivedi M., Laddha N.C., Begum R., Hardikar A.A., Ramachandran A.V. (2012). Oreocnide Integrifolia Flavonoids Augment Reprogramming for Islet Neogenesis and β-Cell Regeneration in Pancreatectomized BALB/c Mice. Evid.-Based Complement. Altern. Med..

[79] Yang L., Yao D., Yang H., Wei Y., Peng Y., Ding Y., Shu L. (2016). Puerarin Protects Pancreatic β-Cells in Obese Diabetic Mice via Activation of GLP-1R Signaling. Mol. Endocrinol..

[80] Kobori M., Masumoto S., Akimoto Y., Takahashi Y. (2009). Dietary Quercetin Alleviates Diabetic Symptoms and Reduces Streptozotocin-Induced Disturbance of Hepatic Gene Expression in Mice. Mol. Nutr. Food Res..

[81] Rahimi M., Sajadimajd S., Mahdian Z., Hemmati M., Malekkhatabi P., Bahrami G., Mohammadi B., Miraghaee S., Hatami R., Mansouri K. (2020). Characterization and Anti-Diabetic Effects of the Oligosaccharide Fraction Isolated from Rosa Canina in STZ-Induced Diabetic Rats. Carbohydr. Res..

[82] Soto C., Raya L., Juárez J., Pérez J., González I. (2014). Effect of Silymarin in Pdx-1 Expression and the Proliferation of Pancreatic β-Cells in a Pancreatectomy Model. Phytomedicine.

[83] Damame H., Rooge S., Patil R., Garad C., Arvindekar A. (2022). In Vitro Differentiation of Human Pancreatic Duct-Derived PANC-1 Cells into β-Cell Phenotype Using *Tinospora cordifolia*. Vitr. Cell. Dev. Biol. Anim..

[84] Levine F. (2022). Approaches to Inducing β-Cell Regeneration. Biomedicines.

[85] Jiang W.J., Peng Y.C., Yang K.M. (2018). Cellular Signaling Pathways Regulating β-Cell Proliferation as a Promising Therapeutic Target in the Treatment of Diabetes (Review). Exp. Ther. Med..

[86] Baeyens L., Bonné S., German M.S., Ravassard P., Heimberg H., Bouwens L. (2006). Ngn3 Expression during Postnatal in Vitro Beta Cell Neogenesis Induced by the JAK/STAT Pathway. Cell Death Differ..

[87] Koblas T., Leontovyč I., Zacharovová K., Berková Z., Kříž J., Girman P., Saudek F. (2012). Activation of the Jak/Stat Signalling Pathway by Leukaemia Inhibitory Factor Stimulates Trans-Differentiation of Human Non-Endocrine Pancreatic Cells into Insulin-Producing Cells. Folia Biol..

[88] Shu L., Zien K., Gutjahr G., Oberholzer J., Pattou F., Kerr-Conte J., Maedler K. (2012). TCF7L2 Promotes Beta Cell Regeneration in Human and Mouse Pancreas. Diabetologia.

[89] Minami K., Okuno M., Miyawaki K., Okumachi A., Ishizaki K., Oyama K., Kawaguchi M., Ishizuka N., Iwanaga T., Seino S. (2005). Lineage Tracing and Characterization of Insulin-Secreting Cells Generated from Adult Pancreatic Acinar Cells. Proc. Natl. Acad. Sci. USA.

[90] Elghazi L., Rachdi L., Weiss A.J., Cras-Méneur C., Bernal-Mizrachi E. (2007). Regulation of β-Cell Mass and Function by the Akt/Protein Kinase B Signalling Pathway. Diabetes Obes. Metab..

[91] Sarbassov D.D., Guertin D.A., Ali S.M., Sabatini D.M. (2005). Phosphorylation and Regulation of Akt/PKB by the Rictor-MTOR Complex. Science.

[92] Woodgett J.R. (2005). Recent Advances in the Protein Kinase B Signaling Pathway. Curr. Opin. Cell Biol..

[93] Du K., Tsichlis P.N. (2005). Regulation of the Akt Kinase by Interacting Proteins. Oncogene.

[94] Aguirre V., Werner E.D., Giraud J., Lee Y.H., Shoelson S.E., White M.F. (2002). Phosphorylation of Ser307 in Insulin Receptor Substrate-1 Blocks Interactions with the Insulin Receptor and Inhibits Insulin Action. J. Biol. Chem..

[95] Kaneto H., Matsuoka T., Nakatani Y., Kawamori D., Matsuhisa M., Yamasaki Y. (2005). Oxidative Stress and the JNK Pathway in Diabetes. Curr. Diabetes Rev..

[96] Ozcan U., Cao Q., Yilmaz E., Lee A.-H., Iwakoshi N.N., Ozdelen E., Tuncman G., Gorgun C., Glimcher L.H., Hotamisligil G.S. (2016). Endoplasm. Encyclopedia of Parasitology.

[97] Lanuza-Masdeu J., Isabel Arévalo M., Vila C., Barberà A., Gomis R., Caelles C. (2013). In Vivo Jnk Activation in Pancreatic β-Cells Leads to Glucose Intolerance Caused by Insulin Resistance in Pancreas. Diabetes.

[98] Um S.H., D’Alessio D., Thomas G. (2006). Nutrient Overload, Insulin Resistance, and Ribosomal Protein S6 Kinase 1, S6K1. Cell Metab..

[99] Buteau J., Foisy S., Joly E., Prentki M. (2003). Glucagon-like Peptide 1 Induces Pancreatic β-Cell Proliferation via Transactivation of the Epidermal Growth Factor Receptor. Diabetes.

[100] Jhala U.S., Canettieri G., Screaton R.A., Kulkarni R.N., Krajewski S., Reed J., Walker J., Lin X., White M., Montminy M. (2003). CAMP Promotes Pancreatic β-Cell Survival via CREB-Mediated Induction of IRS2. Genes Dev..

[101] Park S., Dong X., Fisher T.L., Dunn S., Omer A.K., Weir G., White M.F. (2006). Exendin-4 Uses Irs2 Signaling to Mediate Pancreatic β Cell Growth and Function. J. Biol. Chem..

[102] Jetton T.L., Liu Y.Q., Trotman W.E., Nevin P.W., Sun X.J., Leahy J.L. (2001). Enhanced Expression of Insulin Receptor Substrate–2 and Activation of Protein Kinase B/Akt in Regenerating Pancreatic Duct Epithelium of 60%-Partial Pancreatectomy Rats. Diabetologia.

[103] Avruch J., Hara K., Lin Y., Liu M., Long X., Ortiz-Vega S., Yonezawa K. (2006). Insulin and Amino-Acid Regulation of MTOR Signaling and Kinase Activity through the Rheb GTPase. Oncogene.

[104] Hay N., Sonenberg N. (2004). Upstream and Downstream of MTOR. Genes Dev..

[105] Huang J., Dibble C.C., Matsuzaki M., Manning B.D. (2008). The TSC1-TSC2 Complex Is Required for Proper Activation of MTOR Complex 2. Mol. Cell. Biol..

[106] Balcazar N., Sathyamurthy A., Elghazi L., Gould A., Weiss A., Shiojima I., Walsh K., Bernal-Mizrachi E. (2009). MTORC1 Activation Regulates β-Cell Mass and Proliferation by Modulation of Cyclin D2 Synthesis and Stability. J. Biol. Chem..

[107] Kwon G., Marshall C.A., Pappan K.L., Remedi M.S., McDaniel M.L. (2004). Signaling Elements Involved in the Metabolic Regulation of MTOR by Nutrients, Incretins, and Growth Factors in Islets. Diabetes.

[108] Rachdi L., Balcazar N., Osorio-Duque F., Elghazi L., Weiss A., Gould A., Chang-Chen K.J., Gambello M.J., Bernal-Mizrachi E. (2008). Disruption of Tsc2 in Pancreatic β Cells Induces β Cell Mass Expansion and Improved Glucose Tolerance in a TORC1-Dependent Manner. Proc. Natl. Acad. Sci. USA.

[109] Shigeyama Y., Kobayashi T., Kido Y., Hashimoto N., Asahara S., Matsuda T., Takeda A., Inoue T., Shibutani Y., Koyanagi M. (2008). Biphasic Response of Pancreatic β-Cell Mass to Ablation of Tuberous Sclerosis Complex 2 in Mice. Mol. Cell. Biol..

[110] Dos D.S., Ali S.M., Kim D.H., Guertin D.A., Latek R.R., Erdjument-Bromage H., Tempst P., Sabatini D.M. (2004). Rictor, a Novel Binding Partner of MTOR, Defines a Rapamycin-Insensitive and Raptor-Independent Pathway That Regulates the Cytoskeleton. Curr. Biol..

[111] Jara M.A., Werneck-De-Castro J.P., Lubaczeuski C., Johnson J.D., Bernal-Mizrachi E. (2020). Pancreatic and Duodenal Homeobox-1 (PDX1) Contributes to β-Cell Mass Expansion and Proliferation Induced by Akt/PKB Pathway. Islets.

[112] Fatrai S., Elghazi L., Balcazar N., Cras-Méneur C., Krits I., Kiyokawa H., Bernal-Mizrachi E. (2006). Akt Induces β-Cell Proliferation by Regulating Cyclin D1, Cyclin D2, and P21 Levels and Cyclin-Dependent Kinase-4 Activity. Diabetes.

[113] Blandino-Rosano M., Alejandro E.U., Sathyamurthy A., Scheys J.O., Gregg B., Chen A.Y., Rachdi L., Weiss A., Barker D.J., Gould A.P. (2012). Enhanced Beta Cell Proliferation in Mice Overexpressing a Constitutively Active Form of Akt and One Allele of P21Cip. Diabetologia.

[114] Wong J.C., Vo V., Gorjala P., Fiscus R.R. (2017). Pancreatic-β-Cell Survival and Proliferation Are Promoted by Protein Kinase G Type Iα and Downstream Regulation of AKT/FOXO1. Diabetes Vasc. Dis. Res..

[115] You H., Laychock S.G. (2009). Atrial Natriuretic Peptide Promotes Pancreatic Islet β-Cell Growth and Akt/Foxo1a/Cyclin D2 Signaling. Endocrinology.

[116] Wrede C.E., Dickson L.M., Lingohr M.K., Briaud I., Rhodes C.J. (2002). Protein Kinase B/Akt Prevents Fatty Acid-Induced Apoptosis in Pancreatic β-Cells (INS-1). J. Biol. Chem..

[117] Srinivasan S., Ohsugi M., Liu Z., Fatrai S., Bernal-Mizrachi E., Permutt M.A. (2005). Endoplasmic Reticulum Stress-Induced Apoptosis Is Partly Mediated by Reduced Insulin Signaling through Phosphatidylinositol 3-Kinase/Akt and Increased Glycogen Synthase Kinase-3β in Mouse Insulinoma Cells. Diabetes.

[118] Ammendrup A., Maillard A., Nielsen K., Andersen A.N., Serup P., Madsen O.D., Mandrup-Poulsen T., Bonny C. (2000). The C-Jun Amino-Terminal Kinase Pathway Is Preferentially Activated by Interleukin-1 and Controls Apoptosis in Differentiating Pancreatic β-Cells. Diabetes.

[119] Størling J., Binzer J., Andersson A.K., Züllig R.A., Tonnesen M., Lehmann R., Spinas G.A., Sandler S., Billestrup N., Mandrup-Poulsen T. (2005). Nitric Oxide Contributes to Cytokine-Induced Apoptosis in Pancreatic Beta Cells via Potentiation of JNK Activity and Inhibition of Akt. Diabetologia.

[120] Clevers H. (2006). Wnt/β-Catenin Signaling in Development and Disease. Cell.

[121] Mussmann R., Geese M., Harder F., Kegel S., Andag U., Lomow A., Burk U., Onichtchouk D., Dohrmann C., Austen M. (2007). Inhibition of GSK3 Promotes Replication and Survival of Pancreatic Beta Cells. J. Biol. Chem..

[122] Welters H.J., Kulkarni R.N. (2008). Wnt Signaling: Relevance to β-Cell Biology and Diabetes. Trends Endocrinol. Metab..

[123] Rulifson I.C., Karnik S.K., Heiser P.W., Ten Berge D., Chen H., Gu X., Taketo M.M., Nusse R., Hebrok M., Kim S.K. (2007). Wnt Signaling Regulates Pancreatic Beta Cell Proliferation. Proc. Natl. Acad. Sci. USA.

[124] Heiser P.W., Lau J., Taketo M.M., Herrera P.L., Hebrok M. (2006). Stabilization of β-Catenin Impacts Pancreas Growth. Development.

[125] Rane S.G., Reddy E.P. (2000). Cell Cycle Control of Pancreatic Beta Cell Proliferation. Front. Biosci..

[126] El-Badawy A., El-Badri N. (2016). The Cell Cycle as a Brake for β-Cell Regeneration from Embryonic Stem Cells. Stem Cell Res. Ther..

[127] Ohsugi M., Gras-Méneur C., Zhou Y., Bernal-Mizrachi E., Johnson J.D., Luciani D.S., Polonsky K.S., Permutt M.A. (2005). Reduced Expression of the Insulin Receptor in Mouse Insulinoma (MIN6) Cells Reveals Multiple Roles of Insulin Signaling in Gene Expression, Proliferation, Insulin Content, and Secretion. J. Biol. Chem..

[128] Tanabe K., Liu Z., Patel S., Doble B.W., Li L., Cras-Méneur C., Martinez S.C., Welling C.M., White M.F., Bernal-Mizrachi E. (2008). Genetic Deficiency of Glycogen Synthase Kinase-3β Corrects Diabetes in Mouse Models of Insulin Resistance. PLoS Biol..

[129] Boucher M.J., Selander L., Carlsson L., Edlund H. (2006). Phosphorylation Marks IPF1/PDX1 Protein for Degradation by Glycogen Synthase Kinase 3-Dependent Mechanisms. J. Biol. Chem..

[130] Chang F., Lee J.T., Navolanic P.M., Steelman L.S., Shelton J.G., Blalock W.L., Franklin R.A., McCubrey J.A. (2003). Involvement of PI3K/Akt Pathway in Cell Cycle Progression, Apoptosis, and Neoplastic Transformation: A Target for Cancer Chemotherapy. Leukemia.

[131] Kitamura T., Nakae J., Kitamura Y., Kido Y., Biggs W.H., Wright C.V.E., White M.F., Arden K.C., Accili D. (2002). The Forkhead Transcription Factor Foxo1 Links Insulin Signaling to Pdx1 Regulation of Pancreatic β Cell Growth. J. Clin. Investig..

[132] Hashimoto N., Kido Y., Uchida T., Asahara S.I., Shigeyama Y., Matsuda T., Takeda A., Tsuchihashi D., Nishizawa A., Ogawa W. (2006). Ablation of PDK1 in Pancreatic β Cells Induces Diabetes as a Result of Loss of β Cell Mass. Nat. Genet..

[133] Nakamura K., Sakaue H., Nishizawa A., Matsuki Y., Gomi H., Watanabe E., Hiramatsu R., Tamamori-Adachi M., Kitajima S., Noda T. (2008). PDK1 Regulates Cell Proliferation and Cell Cycle Progression through Control of Cyclin D1 and P27Kip1 Expression. J. Biol. Chem..

[134] Nakae J., Oki M., Cao Y. (2008). The FoxO Transcription Factors and Metabolic Regulation. FEBS Lett..

[135] Okamoto H., Hribal M.L., Lin H.V., Bennett W.R., Ward A., Accili D. (2006). Role of the Forkhead Protein FoxO1 in β Cell Compensation to Insulin Resistance. J. Clin. Investig..

[136] Buteau J., Spatz M.L., Accili D. (2006). Transcription Factor FoxO1 Mediates Glucagon-like Peptide-1 Effects on Pancreatic β-Cell Mass. Diabetes.

[137] Martinez S.C., Cras-Méneur C., Bernal-Mizrachi E., Permutt M.A. (2006). Glucose Regulates Foxo1 through Insulin Receptor Signaling in the Pancreatic Islet β-Cell. Diabetes.

[138] Pende M., Kozma S.C., Jaquet M., Oorschot V., Burcelin R., Le Marchand-Brustel Y., Klumperman J., Thorens B., Thomas G. (2000). Hypoinsulinaemia, Glucose Intolerance and Diminished β-Cell Size in S6K1-Deficient Mice. Nature.

[139] Bernal-Mizrachi E., Wen W., Stahlhut S., Welling C.M., Permutt M.A. (2001). Islet Beta Cell Expression of Constitutively Active Akt1/PKB Alpha Induces Striking Hypertrophy, Hyperplasia, and Hyperinsulinemia. J. Clin. Investig..

[140] Rane S.G., Lee J.H., Lin H.M. (2006). Transforming Growth Factor-β Pathway: Role in Pancreas Development and Pancreatic Disease. Cytokine Growth Factor Rev..

[141] El-Gohary Y., Tulachan S., Guo P., Welsh C., Wiersch J., Prasadan K., Paredes J., Shiota C., Xiao X., Wada Y. (2013). Smad Signaling Pathways Regulate Pancreatic Endocrine Development. Dev. Biol..

[142] Jiang Y., Fischbach S., Xiao X. (2018). The Role of the TGFβ Receptor Signaling Pathway in Adult Beta Cell Proliferation. Int. J. Mol. Sci..

[143] Hanley S., Rosenberg L. (2007). Transforming Growth Factor β Is a Critical Regulator of Adult Human Islet Plasticity. Mol. Endocrinol..

[144] Feng X.H., Derynck R. (2005). Specificity and Versatility in TGF-β Signaling through Smads. Annu. Rev. Cell Dev. Biol..

[145] Sjoholm A., Hellerstrom C. (1991). TGF-β Stimulates Insulin Secretion and Blocks Mitogenic Response of Pancreatic β-Cells to Glucose. Am. J. Physiol.-Cell Physiol..

[146] Smart N.G., Apelqvist Å.A., Gu X., Harmon E.B., Topper J.N., MacDonald R.J., Kim S.K. (2006). Conditional Expression of Smad7 in Pancreatic β Cells Disrupts TGF-β Signaling and Induces Reversible Diabetes Mellitus. PLoS Biol..

[147] Toren-Haritan G., Efrat S. (2015). TGFβ Pathway Inhibition Redifferentiates Human Pancreatic Islet β Cells Expanded In Vitro. PLoS ONE.

[148] Matsumura H., Kudo T., Harada A., Esaki R., Suzuki H., Kato M., Takahashi S. (2007). Suppression of MafA-Dependent Transcription by Transforming Growth Factor-β Signaling. Biochem. Biophys. Res. Commun..

[149] Lin H.M., Lee J.H., Yadav H., Kamaraju A.K., Liu E., Zhigang D., Vieira A., Kim S.J., Collins H., Matschinsky F. (2009). Transforming Growth Factor-β/Smad3 Signaling Regulates Insulin Gene Transcription and Pancreatic Islet β-Cell Function. J. Biol. Chem..

[150] Suzuki T., Dai P., Hatakeyama T., Harada Y., Tanaka H., Yoshimura N., Takamatsu T. (2013). TGF-β Signaling Regulates Pancreatic β-Cell Proliferation through Control of Cell Cycle Regulator P27 Expression. Acta Histochem. Cytochem..

[151] Sehrawat A., Shiota C., Mohamed N., DiNicola J., Saleh M., Kalsi R., Zhang T., Wang Y., Prasadan K., Gittes G.K. (2020). SMAD7 Enhances Adult β-Cell Proliferation without Significantly Affecting β-Cell Function in Mice. J. Biol. Chem..

[152] Blokzijl A., Dahlqvist C., Reissmann E., Falk A., Moliner A., Lendahl U., Ibáñez C.F. (2003). Cross-Talk between the Notch and TGF-β Signaling Pathways Mediated by Interaction of the Notch Intracellular Domain with Smad3. J. Cell Biol..

[153] Bartolome A., Zhu C., Sussel L., Pajvani U.B. (2019). Notch Signaling Dynamically Regulates Adult β Cell Proliferation and Maturity. J. Clin. Investig..

[154] El-Gohary Y., Tulachan S., Wiersch J., Guo P., Welsh C., Prasadan K., Paredes J., Shiota C., Xiao X., Wada Y. (2014). A Smad Signaling Network Regulates Islet Cell Proliferation. Diabetes.

[155] Xiao X., Wiersch J., El-Gohary Y., Guo P., Prasadan K., Paredes J., Welsh C., Shiota C., Gittes G.K. (2013). TGFβ Receptor Signaling Is Essential for Inflammation-Induced but Not β-Cell Workload-Induced β-Cell Proliferation. Diabetes.

[156] Xiao X., Gaffar I., Guo P., Wiersch J., Fischbach S., Peirish L., Song Z., El-Gohary Y., Prasadan K., Shiota C. (2014). M2 Macrophages Promote Beta-Cell Proliferation by up-Regulation of SMAD7. Proc. Natl. Acad. Sci. USA.

[157] Hayes H.L., Zhang L., Becker T.C., Haldeman J.M., Stephens S.B., Arlotto M., Moss L.G., Newgard C.B., Hohmeier H.E. (2016). A Pdx-1-Regulated Soluble Factor Activates Rat and Human Islet Cell Proliferation. Mol. Cell. Biol..

[158] Wang P., Karakose E., Liu H., Swartz E., Zlatanic V., Wilson J., González B.J., Takane K.K., Ye L., Harb G. (2019). Combined Inhibition of DYRK1A, SMAD and Trithorax Pathways Synergizes to Induce Robust Replication in Adult Human Beta Cells. Cell Metab..

[159] Drucker D.J., Nauck M.A. (2006). The Incretin System: Glucagon-like Peptide-1 Receptor Agonists and Dipeptidyl Peptidase-4 Inhibitors in Type 2 Diabetes. Lancet.

[160] Buteau J., Roduit R., Susini S., Prentki M. (1999). Glucagon-like Peptide-1 Promotes DNA Synthesis, Activates Phosphatidylinositol 3-Kinase and Increases Transcription Factor Pancreatic and Duodenal Homeobox Gene 1 (PDX-1) DNA Binding Activity in Beta (INS-1)- Cells. Diabetologia.

[161] Buteau J., Foisy S., Rhodes C.J., Carpenter L., Biden T.J., Prentki M. (2001). Protein Kinase Czeta Activation Mediates Glucagon-like Peptide-1-Induced Pancreatic Beta-Cell Proliferation. Diabetes.

[162] Furukawa N., Shirotani T., Araki E., Kaneko K., Todaka M., Matsumoto K., Tsuruzoe K., Motoshima H., Yoshizato K., Kishikawa H. (1999). Possible Involvement of Atypical Protein Kinase C (PKC) in Glucose- Sensitive Expression of the Human Insulin Gene: DNA-Binding Activity and Transcriptional Activity of Pancreatic and Duodenal Homeobox Gene-1 (PDX-1) Are Enhanced via Calphostin C-Sensitiv. Endocr. J..

[163] Wang Q., Li L., Xu E., Wong V., Rhodes C., Brubaker P.L. (2004). Glucagon-like Peptitle-1 Regulates Proliferation and Apoptosis via Activation of Protein Kinase B in Pancreatic INS-1 Beta Cells. Diabetologia.

[164] Wang C., Chen X., Ding X., He Y., Gu C., Zhou L. (2015). Exendin-4 Promotes Beta Cell Proliferation via PI3k/Akt Signalling Pathway. Cell. Physiol. Biochem..

[165] Stoffers D.A., Kieffer T.J., Hussain M.A., Drucker D.J., Bonner-Weir S., Habener J.F., Egan J.M. (2000). Insulinotropic Glucagon-like Peptide 1 Agonists Stimulate Expression of Homeodomain Protein IDX-1 and Increase Islet Size in Mouse Pancreas. Diabetes.

[166] Wang Q., Brubaker P. (2002). Glucagon-like Peptide-1 Treatment Delays the Onset of Diabetes in 8 Week-Old Db/Db Mice. Diabetologia.

[167] De León D.D., Deng S., Madani R., Ahima R.S., Drucker D.J., Stoffers D.A. (2003). Role of Endogenous Glucagon-like Peptide-1 in Islet Regeneration after Partial Pancreatectomy. Diabetes.

[168] Xu G., Stoffers D.A., Habener J.F., Bonner-Weir S. (1999). Exendin-4 Stimulates Both Beta-Cell Replication and Neogenesis, Resulting in Increased Beta-Cell Mass and Improved Glucose Tolerance in Diabetic Rats. Diabetes.

[169] Tourrel C., Bailbé D., Meile M.J., Kergoat M., Portha B. (2001). Glucagon-like Peptide-1 and Exendin-4 Stimulate Beta-Cell Neogenesis in Streptozotocin-Treated Newborn Rats Resulting in Persistently Improved Glucose Homeostasis at Adult Age. Diabetes.

[170] Murtaugh L.C. (2008). The What, Where, When and How of Wnt/β-Catenin Signaling in Pancreas Development. Organogenesis.

[171] Baek S.H., Kioussi C., Briata P., Wang D., Nguyen H.D., Ohgi K.A., Glass C.K., Wynshaw-Boris A., Rose D.W., Rosenfeld M.G. (2003). Regulated Subset of G1 Growth-Control Genes in Response to Derepression by the Wnt Pathway. Proc. Natl. Acad. Sci. USA.

[172] Briata P., Ilengo C., Corte G., Moroni C., Rosenfeld M.G., Chen C.-Y., Gherzi R. (2003). The Wnt/β-Catenin→Pitx2 Pathway Controls the Turnover of Pitx2 and Other Unstable MRNAs. Mol. Cell.

[173] Liu Z., Habener J.F. (2008). Glucagon-like Peptide-1 Activation of TCF7L2-Dependent Wnt Signaling Enhances Pancreatic Beta Cell Proliferation. J. Biol. Chem..

[174] Hino S., Tanji C., Nakayama K.I., Kikuchi A. (2005). Phosphorylation of β-Catenin by Cyclic AMP-Dependent Protein Kinase Stabilizes β-Catenin through Inhibition of Its Ubiquitination. Mol. Cell. Biol..

[175] Heller C., Kühn M.C., Mülders-Opgenoorth B., Schott M., Willenberg H.S., Scherbaum W.A., Schinner S. (2011). Exendin-4 Upregulates the Expression of Wnt-4, a Novel Regulator of Pancreatic β-Cell Proliferation. Am. J. Physiol.-Endocrinol. Metab..

[176] Gui S., Yuan G., Wang L., Zhou L., Xue Y., Yu Y., Zhang J., Zhang M., Yang Y., Wang D.W. (2013). Wnt3a Regulates Proliferation, Apoptosis and Function of Pancreatic NIT-1 Beta Cells via Activation of IRS2/PI3K Signaling. J. Cell. Biochem..

[177] Maschio D.A., Oliveira R.B., Santos M.R., Carvalho C.P.F., Barbosa-Sampaio H.C.L., Collares-Buzato C.B. (2016). Activation of the Wnt/β-Catenin Pathway in Pancreatic Beta Cells during the Compensatory Islet Hyperplasia in Prediabetic Mice. Biochem. Biophys. Res. Commun..

[178] Figeac F., Uzan B., Faro M., Chelali N., Portha B., Movassat J. (2010). Neonatal Growth and Regeneration of β-Cells Are Regulated by the Wnt/β-Catenin Signaling in Normal and Diabetic Rats. Am. J. Physiol.-Endocrinol. Metab..

[179] Tsukiyama S., Matsushita M., Matsumoto S., Morita T., Kobayashi S., Tamura H., Kamachi H., Ozaki M., Todo S. (2006). Transduction of Exogenous Constitutively Activated Stat3 into Dispersed Islets Induces Proliferation of Rat Pancreatic β-Cells. Tissue Eng..

[180] Levy D.E., Darnell J.E. (2002). STATs: Transcriptional Control and Biological Impact. Nat. Rev. Mol. Cell Biol..

[181] Valdez I.A., Dirice E., Gupta M.K., Shirakawa J., Teo A.K.K., Kulkarni R.N. (2016). Proinflammatory Cytokines Induce Endocrine Differentiation in Pancreatic Ductal Cells via STAT3-Dependent NGN3 Activation. Cell Rep..

[182] Yamauchi A., Itaya-Hironaka A., Sakuramoto-Tsuchida S., Takeda M., Yoshimoto K., Miyaoka T., Fujimura T., Tsujinaka H., Tsuchida C., Ota H. (2015). Synergistic Activations of REG i α and REG i β Promoters by IL-6 and Glucocorticoids through JAK/STAT Pathway in Human Pancreatic β Cells. J. Diabetes Res..

[183] Miura M., Miyatsuka T., Katahira T., Sasaki S., Suzuki L., Himuro M., Nishida Y., Fujitani Y., Matsuoka T.-A., Watada H. (2018). Suppression of STAT3 Signaling Promotes Cellular Reprogramming into Insulin-Producing Cells Induced by Defined Transcription Factors. EBioMedicine.

[184] Baeyens L., Lemper M., Staels W., De Groef S., De Leu N., Heremans Y., German M.S., Heimberg H. (2018). (Re)Generating Human Beta Cells: Status, Pitfalls, and Perspectives. Physiol. Rev..

[185] Su G.Y., Wang K.W., Wang X.Y., Wu B. (2015). Bioactive Lignans from Zanthoxylum Planispinum with Cytotoxic Potential. Phytochem. Lett..

[186] Afelik S., Rovira M. (2017). Pancreatic β-Cell Regeneration: Advances in Understanding the Genes and Signaling Pathways Involved. Genome Med..

[187] Domínguez-Bendala J., Qadir M.M.F., Pastori R.L. (2019). Pancreatic Progenitors: There and Back Again. Trends Endocrinol. Metab..

[188] Nir T., Melton D.A., Dor Y. (2007). Recovery from Diabetes in Mice by β Cell Regeneration. J. Clin. Investig..

[189] Teta M., Rankin M.M., Long S.Y., Stein G.M., Kushner J.A. (2007). Growth and Regeneration of Adult Beta Cells Does Not Involve Specialized Progenitors. Dev. Cell.

[190] Lee C.S., De León D.D., Kaestner K.H., Stoffers D.A. (2006). Regeneration of Pancreatic Islets after Partial Pancreatectomy in Mice Does Not Involve the Reactivation of Neurogenin-3. Diabetes.

[191] Thorel F., Népote V., Avril I., Kohno K., Desgraz R., Chera S., Herrera P.L. (2010). Conversion of Adult Pancreatic α-Cells to β-Cells after Extreme β-Cell Loss. Nature.

[192] Misfeldt A.A., Costa R.H., Gannon M. (2008). Beta-Cell Proliferation, but Not Neogenesis, Following 60% Partial Pancreatectomy Is Impaired in the Absence of FoxM1. Diabetes.

[193] Hayashi K.Y., Tamaki H., Handa K., Takahashi T., Kakita A., Yamashina S. (2003). Differentiation and Proliferation of Endocrine Cells in the Regenerating Rat Pancreas after 90% Pancreatectomy. Arch. Histol. Cytol..

[194] Li W.C., Rukstalis J.M., Nishimura W., Tchipashvili V., Habener J.F., Sharma A., Bonner-Weir S. (2010). Activation of Pancreatic-Duct-Derived Progenitor Cells during Pancreas Regeneration in Adult Rats. J. Cell Sci..

[195] Bonner-Weir S., Baxter L.A., Schuppin G.T., Smith F.E. (1993). A Second Pathway for Regeneration of Adult Exocrine and Endocrine Pancreas: A Possible Recapitulation of Embryonic Development. Diabetes.

[196] Zhang M., Lin Q., Qi T., Wang T., Chen C.C., Riggs A.D., Zeng D. (2016). Growth Factors and Medium Hyperglycemia Induce Sox9+ Ductal Cell Differentiation into β Cells in Mice with Reversal of Diabetes. Proc. Natl. Acad. Sci. USA.

[197] Cai E.P., Lin J.-K. (2009). Epigallocatechin Gallate (EGCG) and Rutin Suppress the Glucotoxicity through Activating IRS2 and AMPK Signaling in Rat Pancreatic β Cells. J. Agric. Food Chem..

[198] Prentki M., Nolan C.J. (2006). Islet β-Cell Failure in Type 2 Diabetes. J. Clin. Investig..

[199] Eguchi N., Vaziri N.D., Dafoe D.C., Ichii H. (2021). The Role of Oxidative Stress in Pancreatic β Cell Dysfunction in Diabetes. Int. J. Mol. Sci..

[200] Böni-Schnetzler M., Meier D.T. (2019). Islet Inflammation in Type 2 Diabetes. Semin. Immunopathol..

[201] Kanatsuka A., Kou S., Makino H. (2018). IAPP/Amylin and β-Cell Failure: Implication of the Risk Factors of Type 2 Diabetes. Diabetol. Int..

[202] Leibowitz G., Kaiser N., Cerasi E. (2011). β-Cell Failure in Type 2 Diabetes. J. Diabetes Investig..

[203] Cernea S., Dobreanu M. (2013). Diabetes and Beta Cell Function: From Mechanisms to Evaluation and Clinical Implications. Biochem. Medica.

[204] Nair G., Hebrok M. (2015). Islet Formation in Mice and Men: Lessons for the Generation of Functional Insulin-Producing β Cells from Human Pluripotent Stem Cells. Curr. Opin. Genet. Dev..

[205] Levetan C.S., Pierce S.M. (2013). Distinctions Between the Islets of Mice and Men: Implications for New Therapies for Type 1 and 2 Diabetes. Endocr. Pract..

